# The Mirror to Our Soul? Comparisons of Spontaneous and Posed Vocal Expression of Emotion

**DOI:** 10.1007/s10919-017-0268-x

**Published:** 2017-10-25

**Authors:** Patrik N. Juslin, Petri Laukka, Tanja Bänziger

**Affiliations:** 1 Department of Psychology, Uppsala University, Box 1225, 751 42 Uppsala, Sweden; 2 Department of Psychology, Stockholm University, Stockholm, Sweden

**Keywords:** Communication, Emotion, Expression, Nonverbal, Voice

## Abstract

It has been the subject of much debate in the study of vocal expression of emotions whether posed expressions (e.g., actor portrayals) are different from spontaneous expressions. In the present investigation, we assembled a new database consisting of 1877 voice clips from 23 datasets, and used it to systematically compare spontaneous and posed expressions across 3 experiments. Results showed that (a) spontaneous expressions were generally rated as more genuinely emotional than were posed expressions, even when controlling for differences in emotion intensity, (b) there were differences between the two stimulus types with regard to their acoustic characteristics, and (c) spontaneous expressions with a high emotion intensity conveyed discrete emotions to listeners to a similar degree as has previously been found for posed expressions, supporting a dose–response relationship between intensity of expression and discreteness in perceived emotions. Our conclusion is that there are reliable differences between spontaneous and posed expressions, though not necessarily in the ways commonly assumed. Implications for emotion theories and the use of emotion portrayals in studies of vocal expression are discussed.

## Introduction

It is commonly believed by lay people that nonverbal cues in the voice reveal our inner emotions to a listener. But does the voice convey specific emotions in real life? Or is it only when actors portray emotions in a stereotypical manner that each emotion is given a distinct voice profile? In this investigation, we compare spontaneous and posed vocal expressions to examine whether they are truly different.

### Studies of Vocal Expression

The human voice has been called “the mirror to our soul” (Sundberg 1998). Virtually every day of our lives, we make inferences about other individuals’ emotions based on how their voice sounds, often without being aware of doing so (e.g., Pell and Skorup 2008). Most studies of nonverbal communication have focused on the face (Ekman 1973). Yet, findings indicate that relying on voice cues (e.g., voice pitch, speech rate) may be the most common way to infer other people’s emotion states in everyday life (Planalp 1998).

Such inferences are far from perfect, but they are valid often enough to make our social life easier. If we can infer another speaker’s emotions, we may also be able to understand and predict his or her behavior (Plutchik 1994). Our own emotional expression may, in turn, serve to influence that person’s behavior (Krebs and Davies 1993). Hence, expression of emotions is at the core of social organization (Buck 2014).

It may thus come as something of a surprise that it is still debated whether the voice conveys discrete emotions to listeners. Attempts to find emotion-specific patterns of voice cues have been only partially successful, and have tended to produce inconsistent findings (Cowie et al. 2001; Frick 1985; Juslin and Laukka 2003; Murray and Arnott 1993; Scherer 1986). For example, in several studies speech rate increases in joy expressions; in others it decreases. Although this inconsistency can be due to a number of factors (for a discussion, see Juslin and Scherer 2005, pp. 82–83), a commonly proposed explanation is that the voice does not actually convey discrete emotions, but merely the activity dimension of emotions (Davitz 1964) or some combination of arousal and valence (Bachorowski 1999).

However, if this hypothesis is correct, how could we explain that a number of studies have reported a fair degree of emotion differentiation in voice cues (Banse and Scherer 1996; Juslin and Laukka 2001; van Bezooijen 1984)? The most common argument is that previous results are due to a methodological artifact. Those studies of vocal expression that obtained evidence of emotion-specific voice patterns tended to use actor portrayals, and portrayals of emotion in the laboratory could differ from naturally occurring vocal expressions in real life. The use of emotion portrayals has thus been criticized by some researchers (Douglas-Cowie et al. 2003; Kappas and Hess 1995; Owren and Bachorowski 2007).

### Spontaneous Versus Posed Expression

At the heart of the criticism of using portrayals to study vocal expression of emotion is the distinction between *spontaneous* and *posed* vocal expression (e.g., Zuckerman et al. 1979 see also Buck 2014), which has been much discussed in philosophy and pragmatics (Caffi and Janney 1994). On the one hand, a vocal expression may reflect a genuinely felt emotion, with little or no attempt to regulate the expression according to display rules (Ekman and Friesen 1969) and strategic aims (e.g., self-presentation Banse and Scherer 1996). On the other hand, the expression may reflect an intention–whether implicit or explicit–to convey a specific emotion, even though no such emotion is actually felt by the speaker (Fridlund 1994).

We believe that the distinction is a matter of degree, in so far as vocal expressions in everyday life will often contain both spontaneous and posed aspects (Juslin 2013; Scherer 2013). Nonetheless, it seems fair to assume that emotion portrayals primarily reflect posed expression (cf. Wilting et al. 2006), whereas at least *some* proportion of the expressions that occur in everyday life reflect mainly spontaneous expression (Juslin 2013). Instead of arguing about which type of vocal expression is more “natural” than the other, we submit that the distinction should be conceptualized in terms of the degree to which a vocal expression reflects felt emotion or not.

It may be difficult to separate spontaneous and posed aspects of expression in practice (for a recent discussion, see Scherer and Bänziger 2010)–but this should not lead us to think that the distinction is unimportant or meaningless. It *is* a fair charge that the common usage of portrayals is problematic, *if* they differ from spontaneous expressions and *if* the goal is to investigate how real emotions are expressed in the voice. (This was certainly the goal of our own studies that relied on portrayals; see Juslin and Laukka 2001.) If spontaneous and posed expressions differ, this could explain why principles derived from studies of portrayals have not worked well in practical applications (e.g., automatic emotion classification of everyday speech; see Schuller et al. 2011).

Many researchers have tended to assume that emotion portrayals are similar to, and in fact based on, spontaneous expressions (e.g., Banse and Scherer 1996; Davitz 1964; Juslin and Laukka 2001). Others argue that portrayals may be more “intense” and “stereotypical” than spontaneous expressions (Wilting et al. 2006), and may involve “over-acting” (Jürgens et al. 2011). In addition, emotion portrayals typically lack a social context (e.g., on-going dialogue) and can be expected to involve more “reading” of the verbal content (Douglas-Cowie et al. 2003). Finally, portrayals recorded in a laboratory will have a better sound quality than field recordings of naturally occurring speech (Frank et al. 2005). Arguably, most of the above problems could be addressed in terms of the specific research design used. The main problem is whether actors are really able to simulate the precise voice patterns that occur in spontaneous expressions of emotion (whether they are discrete or not). How similar are posed expressions to spontaneous ones?

### Preliminary Comparisons

It needs emphasizing that a comparison of spontaneous and posed expression should be divided into at least two questions: (1) Are the two types of expression *perceptually* different such that listeners can generally discriminate reliably between the two? (2) Are the two types of expression *acoustically* different such that they may be distinguished based on voice cues? Unfortunately, only a few studies have directly compared posed and spontaneous expressions–whether in terms of perceptual or acoustic similarities.

Starting with perception studies, Audibert et al. (2008) reported that 78% of their subjects were able to discriminate between spontaneous and posed expressions beyond chance level in paired comparisons. Other studies reported that subjects were unable to discriminate play-acted (posed) from authentic (spontaneous) expressions beyond chance level, using posed stimuli from both professional actors (Scheiner and Fischer 2011) and non-actors (Jürgens et al. 2015).

Regarding comparisons at the acoustic level, a couple of investigations concluded that spontaneous and posed samples showed quite similar acoustic patterns for the corresponding emotions, the only difference being that the effects were slightly larger (Williams and Stevens 1972) or smaller (Scherer 2013) for portrayals (see also Juslin 2013, Table 7). Other studies reported differences in voice quality and fundamental-frequency contour (Audibert et al. 2010; Jürgens et al. 2011).

In sum, studies so far have produced mixed results, which are ultimately inconclusive. The different outcomes could in part reflect different methods, or the rather small samples in some of the studies. However, the studies also raise a crucial issue: Considering that any two samples are likely to differ in some way, how different must the two samples be, in order for us to conclude that spontaneous and posed vocal expressions really *are* different? If acoustic analyses of the samples reveal only minor differences in the absolute levels of cues, whereas the *patterns* of cues for specific emotions are fully intact, this is hardly sufficient to show that they are different: such differences can easily be observed even *within* the same stimulus type (e.g., spontaneous) as a result of individual differences between speakers, effects of the verbal content (e.g., different languages), the social context, or the experimental design.

What, then, might be counted as strong evidence for a “real” difference? We argue that to the extent that (a) listeners can clearly discriminate between the two types of voice sample and (b) the two types show different patterns of voice cues for the same emotion, then for all practical purposes, they should be regarded as different. Moreover, we believe that resolving this issue requires the use of a large and representative sample of vocal expressions, in order to even out the confounding effects of extraneous factors.

### Explaining Differences: The Role of Emotion Intensity

One factor that could potentially account for reported differences between spontaneous and posed expression in the above studies is that the design did not control for differences in emotion intensity. Studies based on portrayals have typically focused on high intensity (“full blown emotions”; e.g., Juslin and Laukka 2003), whereas studies based on naturally occurring expressions have typically focused on low intensity (e.g., Douglas-Cowie et al. 1999; Greasley et al. 2000). The latter tendency primarily reflects that the recordings were obtained in contexts (e.g., group discussions) where the voice is regulated in accordance with social norms. Intense expressions might be more common in contexts where individuals are not attempting to (or able to) regulate their expression to the same degree, for instance, in intimate settings and extreme situations (Juslin 2013). Ekman (1997) argues that (spontaneous) facial expressions are most difficult to inhibit or modify when the felt emotion is strong. Several authors acknowledge that emotion intensity may play a role in the reported differences between spontaneous and posed vocal expression (cf. Audibert et al. 2010; Juslin and Scherer 2005; Laukka et al. 2011; Scherer 2013).

What might the consequences be, if the spontaneous and posed expressions investigated thus far differ in emotion intensity? First of all, different levels of intensity (low vs. high) may produce vastly different absolute levels of voice cues. In some cases, differences in voice cues can actually be larger between different intensities of the same emotion than between different emotions with the same intensity (see Juslin and Laukka 2001). Second, differences in intensity might influence the perceived discreteness of the emotions conveyed.

**Fig. 1:**
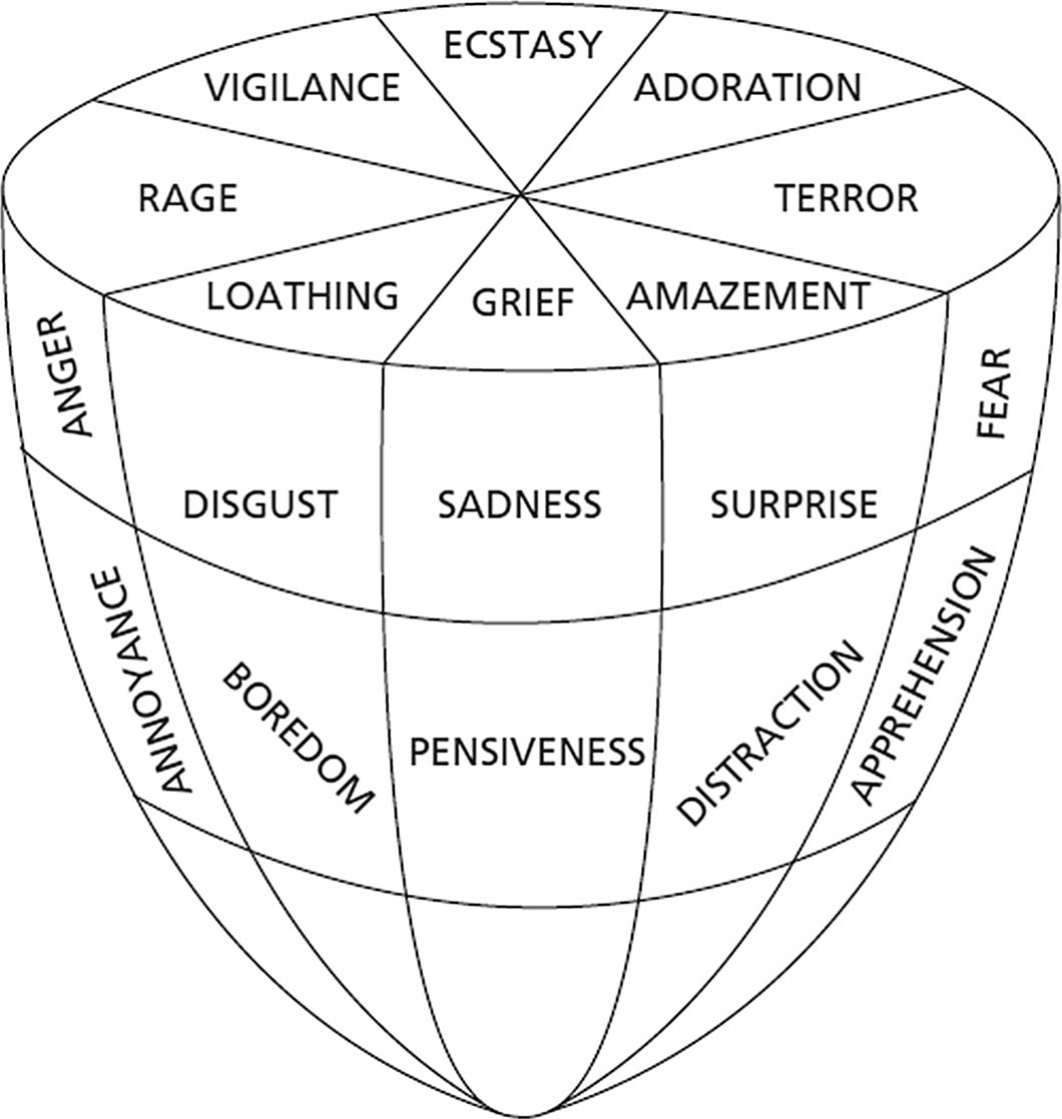
Plutchik’s ‘cone model’ of emotions From Plutchik (1994)

Plutchik (1994) has suggested a structural model of emotions, which has the shape of a cone turned upside down (Fig. 1). The circular structure describes the degree of similarity between emotions, whereas the vertical dimension represents their intensity. Thus, the top of the cone is a neutral center, from which an emotion moves towards a gradually more intense emotion at the bottom. One key implication of this model is that different emotions of a low intensity are more similar to each other than are different emotions of a high intensity; that is, discrete emotions become increasingly different as they get more intense. This could help to explain earlier findings. Specifically, the difficulty in obtaining emotion-specific patterns of voice cues in spontaneous expressions might simply be due to the fact that the samples have featured a low emotion intensity, as compared to most emotion portrayals investigated so far (Juslin 2013).

The above reasoning has two crucial implications: First, it suggests the possibility that spontaneous and posed expression are similar if we control for emotion intensity. Second, it suggests that spontaneous vocal expressions of a high emotion intensity can convey discrete emotions to the same extent as posed expressions (emotion portrayals) with a high intensity.

### Why Does it Matter?

The outcome of the comparison discussed above may have some practical implications for the professional training of interrogators, therapists, and actors, for which the distinction between genuine and feigned emotion is relevant. However, the results also have theoretical implications. At stake are two competing perspectives on emotion. According to categorical theories, people experience emotions as discrete categories such as happiness, sadness, fear, anger, and disgust (Ekman and Cordaro 2011; Izard 1993). In contrast, dimensional theories conceptualize emotions based on their placement along broad affective dimensions, such as arousal and valence (Russell 1980). Failure to obtain emotion-specific patterns of voice cues in recordings of naturally occurring (and presumably spontaneous) vocal expressions can be counted as evidence against categorical approaches—including component-process theories (e.g., Scherer 1986), which typically assume an even greater degree of emotional specificity.

From an evolutionary perspective on vocal expression, however, there are good reasons to assume that spontaneous expressions convey discrete emotions after all (Juslin and Laukka 2003, pp. 771–774). Also, given the lack of studies that involve high-intensity expressions of a spontaneous type, we believe the jury is still out. An evolutionary perspective suggests that vocal expressions commonly occur in situations associated with basic survival problems that various organisms have in common, such as avoiding predators, finding food, competing for resources, and caring for offspring (e.g., Morton 1977; Scherer 1985; Snowdon 2003). The acoustic shape that such vocalizations take reflects physiological reactions which support the emotional behaviors called forth by these urgent events (Levenson 1994). The physiological changes influence many aspects of voice production such as respiration, vocal fold vibration, and articulation (Scherer 1986). From this point of view, then, we would expect spontaneous vocal expressions to convey discrete emotions—at least, if they have a sufficiently high level of emotion intensity.

### Overview of Studies

The aim of this investigation was to compare spontaneous and posed vocal expressions of emotion. Are they perceptually different if one controls for levels of emotion intensity? Do they show different acoustic patterns? Can spontaneous vocal expressions with high intensity convey discrete emotions to the same extent as has been found for posed vocal expressions? As discussed above, the idea was that variation in emotion intensity may help to account for previously observed differences between spontaneous and posed expressions–including the degree of discreteness in perceived emotions.

Douglas-Cowie et al. (2003) noted that “the inherent variability in the area means that to support sound conclusions, databases need to be large” (p. 56). Thus, to address the above questions, we made an effort to obtain a more representative sample of vocal expressions of emotion, than has been used in previous comparisons of spontaneous and posed expressions. This paved the way for a series of studies.

In a pilot study, we evaluate a new database of vocal emotion expressions from which sub-samples are randomly drawn in the following studies. In Study 1, we show that listeners rate spontaneous expressions as more genuinely emotional than posed expressions even after controlling for differences in emotion intensity. In Study 2, we show that there exist acoustic differences among spontaneous and posed expressions, but that the differences are quite few. In Study 3, finally, we demonstrate that spontaneous expressions with high emotion intensity do communicate discrete emotions to listeners, and that there is a dose–response relationship between intensity and discreteness in perceived emotion.

## Pilot Study

### Introduction

The aim of the pilot study was to collect and evaluate a large and representative sample of audio clips featuring spontaneous and posed expressions with both low and high emotion intensity. The resulting database, featuring 1877 clips from 23 sources, was rated by speech experts and lay listeners with respect to emotion intensity, emotional valence, verbal cues to emotions, and recording quality. These ratings were used to compare the databases currently available, eliminate problematic voice clips, and select stimuli for systematic comparisons in the subsequent studies.

### Method

#### Inclusion Criteria

The primary criterion was to include only voice clips consisting of a single grammatical sentence. This appears to be the most frequently used length of voice clips in the field, and for datasets based on portrayals, it is often the only type available. Besides the fact that sentences occur commonly in daily life, they also have the twin advantages of being sufficiently long to contain prosodic contours, still short enough to enable the researcher to include many clips in a single listening test. Previous research has shown that a considerable amount of information is conveyed by an audio recording lasting merely 2 s (Rosenthal 1987). Longer clips (e.g., a dialogue) would not enable us to collect sufficiently large samples of both spontaneous and posed clips, which could be used for comparisons. A focus on single sentences meant that we excluded clips featuring single words (Hawk et al. 2009), pseudo-linguistic contents (Banse and Scherer 1996), and affect bursts such as crying, screams, and laughter (Laukka et al. 2013). Non-linguistic content occurs in some of the selected voice clips, when embedded in longer, linguistically meaningful utterances. Further inclusion criteria were that we only included voice clips featuring healthy adult speakers of a Western language.

#### Search Strategy

To identify potentially available voice recordings, we conducted a literature search of peer-reviewed journal articles published between 2000 and 2013, scanned proceedings from conferences and workshops on emotional corpora (e.g., Affective Computing and Intelligent Interaction), and consulted long lists of databases occurring in El Ayadi et al. (2011), Pittermann et al. (2010), and Ververidis and Kotropoulos (2006).

Forty-five potential datasets were identified, and requests to use the audio material for a novel research application were sent out to the corresponding authors. Some datasets could not be obtained at all–either because authors did not reply to our query or because copyright and privacy restrictions did not allow for sharing of the material. For others, only a subset of the material was available. This was the case with the Belfast Naturalistic Database (Douglas-Cowie et al. 2000) and the HUMAINE database (Douglas-Cowie et al. 2011). A few datasets were also excluded because they lacked emotional annotations (e.g., Carletta 2007; Frommer et al. 2012), or contained noisy recordings (e.g., the SUSAS dataset; Hansen and Bou-Ghazale 1997). We managed to obtain audio recordings from 23 sources that met our criteria for inclusion. Appendix 1 shows a summary of the datasets featured, which comprise five different languages (English, French, German, Dutch, Swedish). (The categorization into spontaneous and posed expressions is based on the labeling by the respective researchers.)

The recordings in the posed datasets were already segmented into voice clips. Emotion portrayals were randomly selected from these datasets, although we constrained the selection so as to be distributed over all speakers, emotions, and sentences present in a specific dataset in order to minimize repetition of the same speaker or the same verbal material. As concerns the spontaneous datasets, some files were already edited, others contained hours of unedited speech. In the latter case, we used the available emotional annotations to identify the speech segments that were most likely to feature emotional information. In cases where the original sound file was not pre-segmented, we manually extracted the relevant segments. A problem in previous attempts to collect spontaneous expressions is that vocal expressions with a high emotion intensity are quite rare (Cowie and Cornelius 2003). Thus, we initially selected more spontaneous clips than posed ones to improve our chances of obtaining enough spontaneous expressions with high intensity. The resulting database included 1877 voice clips that could be rated by listeners.

#### Participants and Procedure

Three senior researchers and speech experts (one female, ages 40–44 years) and three naïve listeners (college students from Stockholm, two females, ages = 24–30 years) took part in the study. The lay participants received monetary compensation for their anonymous and voluntary participation (3000 SEK). None of the participants reported any hearing problem. All 1877 voice clips were rated by each participant. For each clip, he or she was required to rate emotion intensity, valence, verbal cues to emotion, and recording quality in accordance with the following instructions:

##### Emotionality

This refers to the extent to which the person talking sounds emotional or not. The scale ranges from *no emotion* (0) to *much emotion* (4). *No emotion* means that you cannot perceive any emotion at all in the person’s voice. *Much emotion* means that the person sounds like he or she is experiencing a strong (intense) emotion.

##### Valence

This refers to whether it sounds like the person is having a positive (pleasant) feeling or a negative (unpleasant) feeling. The scale ranges from *negative* (− 2) to *positive* (+ 2). If the speaker sounds happy, that would be a case of positive valence, whereas if the speaker sounds sad, that would be a case of negative valence. If the speaker sounds neutral, that would correspond to the middle position (0) of the scale.

##### Verbal Cues

This refers to the extent to which the verbal content (the actual words) of the utterance helps you to infer something about the emotion felt by the speaker. The scale ranges from *no cues* (0) to *strong cues* (4). *No cues* means that nothing in the verbal content conveys any information to you about the emotion felt. *Strong cues* means that the verbal content contains information that strongly implies a certain emotion. (Examples may be emotion words, affectively laden words, or a description of the situation.) If you cannot understand anything of the verbal content (e.g., because the language is foreign), you should simply rate the clip as 0 (*no cues*).

##### Sound Quality

This refers to the perceived acoustic quality of the sound recording as such. The scale ranges from *unacceptable* (0) to *excellent* (4). *Unacceptable* means that the recording is so bad that you can barely hear the nature of the voice. Bad sound quality can be due to noise, perturbations or extraneous sounds interfering with the speaker’s voice. *Excellent* means that the recording is crisp and clear, such that the voice is easy to rate.

The rating tasks were conducted individually, using custom software to present stimuli and collect responses. The participants listened to the recordings using headphones and were allowed to adjust the sound level of the playback if needed. They were also allowed to listen to each recording as many times as required to reach a judgment. (Because some of the clips are very short and can be missed during a momentary lapse of attention, the repeat-playback approach was considered useful to reduce guessing and increase the reliability of the ratings; Scherer and Bänziger 2010; Hawk et al. 2009.) The voice recordings were randomly divided into 19 same-language blocks (eight English, five German, four French, one Swedish, and one Dutch). The order of the blocks, and the order of the stimuli within blocks, were randomized for each participant. The rating of a block took between 30 min and 1 h depending on rater and block. Ratings were done in several consecutive sessions.

### Results and Discussion

Computation of the intra-class correlation coefficient (ICC), using a two-way random effects model and a consistency definition (average measures), indicated that the consistency across the six raters was satisfactory for all four scales: emotion intensity, ICC = .88, *F*(1874, 9370) = 8.34, *p* < .001; valence, ICC = .88, *F*(1874, 9370) = 7.99, *p* < .001; verbal cues, ICC = .80, *F*(1874, 9370) = 5.02, *p* < .001; and sound quality, ICC = .79, *F*(1874, 9370) = 4.75, *p* < .001. Thus, in the following analyses, we use the mean ratings to describe the two stimulus types in the database (spontaneous vs. posed clips).

Ratings of emotion intensity were higher for posed clips (*M* = 1.58, *SD* = 0.82, range: 0.00–4.00) than for spontaneous clips (*M* = 1.28, *SD* = 0.73, range: 0.00–3.83), *t*_1875_ = 8.08, *p* < .001, *d* = 0.39. Posed clips were rated lower in valence (*M* = − 0.47, *SD* = 0.79, range: − 2.00–1.83) than were spontaneous clips (*M* = − 0.25, *SD* = 0.72, range: − 2.00–1.83), *t*_1875_ = − 6.20, *p* < .001, *d* = 0.29. Posed clips were also rated lower in verbal cues (*M* = 0.54, *SD* = 0.83, range: 0.00–3.67) than were spontaneous clips (*M* = 0.65, *SD* = 0.76, range: 0.00–3.50), *t*_1875_ = − 2.82, *p* = .005, *d* = 0.14. Finally, posed clips were rated higher in sound quality (*M* = 2.91, *SD* = 0.55, range: 0.67–3.83) than were spontaneous clips (*M* = 2.75, *SD* = 0.65, range: 0.67–3.67), *t*_1875_ = 5.36, *p* < .001, *d* = 0.25. However, the difference in emotion intensity was the largest.

Averages across all rated stimuli showed that most voice clips included in the database had low emotion intensity (*M* = 1.38, *SD* = 0.77), few verbal cues (*M* = 0.61, *SD* = 0.78), and good sound quality (*M* = 2.80, *SD* = 0.62). Average valence was fairly neutral, but skewed to the negative side (*M* = − 0.32, *SD* = 0.75; on the scale from − 2 to + 2).

The results suggest that spontaneous and posed expressions in the currently available datasets differ in all four of the dimensions judged in the test, although the ranges of ratings reported above indicate that there was considerable variability in these datasets, even within each sample type. In general, the findings confirm that studies have to take these extraneous factors into consideration in order to enable more unbiased comparisons of spontaneous and posed expressions.

## Study 1

### Introduction

The pilot study indicated that the main difference between the available voice clips of spontaneous and posed expressions concerned emotion intensity. Spontaneous clips usually have lower emotion intensity than posed clips, which could potentially account for reported perceptual differences. Accordingly, in order to conduct a “fair” comparison of spontaneous and posed expressions, we have to control for overall differences in emotion intensity in the datasets used. Thus, in Study 1 we utilized a *stratified random sampling procedure* (Visser et al. 2000) to obtain both spontaneous and posed expressions with three levels of emotion intensity (low–medium–high), and required listeners to judge the extent to which they believed each voice clip was an expression of a genuine (spontaneous) emotion. We predicted that the two stimulus-types would not differ significantly in rated spontaneity when controlling for differences in emotion intensity.

### Method

#### Stimulus Material

We used ratings from the pilot study to prepare a smaller set of spontaneous and posed voice clips that were matched concerning emotion intensity. First, we excluded all clips with very poor sound quality, as defined by a mean sound quality rating lower than 1.5 (*N* = 106). Voice clips with inferior sound quality occurred mainly in the spontaneous databases and we were concerned that this factor might bias the comparison. We also excluded clips that were rated as non-emotional, as shown by an emotion-intensity rating smaller than or equal to 1.0 (*N* = 781). Clips without any perceivable emotion can be regarded as irrelevant to the present comparison.

The remaining voice clips were categorized into three categories based on the emotion intensity ratings: “low intensity” (rating > 1 and ≤ 2), “medium intensity” (rating > 2 and ≤ 3), and “high intensity” (rating > 3). From this set (*N* = 990), we then randomly selected 20 clips for each intensity level, for both spontaneous and posed expressions, with the only constraint that no verbal content (e.g., a specific sentence) should occur more than once. This constraint was added, because repetition of the same verbal content could signal to the participant that a voice clip is posed. For posed clips with high intensity, there were only 17 unique sentences available. Hence, the final selection consisted of 117 clips, rather than 120, that were rated by all listeners.

The distribution of selected voice clips across original datasets is shown in Appendix 2. Table 1 shows the mean values for emotion intensity, valence, verbal cues, and sound quality in each condition based on the ratings in the pilot study. Note that the spontaneous and posed samples have fairly similar means overall. The confidence intervals indicate that for medium and high intensity clips, the spontaneous clips featured more verbal cues than the posed clips, but the mean values for the spontaneous clips (1.29 and 1.53) suggest that even they featured few verbal cues, on the whole. Spontaneous and posed clips also differed in sound quality for high intensity clips, but at a generally high level (means > 2.50). Note also that high intensity clips had more negative valence–but this was true for both spontaneous and posed clips. The grand means (bottom row) for verbal cues and sound quality are relatively similar to those for the database as a whole (Pilot Study), whereas the intensity is higher and the valence is lower than in the complete database. These latter data directly reflect the sampling of three intensity levels, because higher intensities involve more negative valence (see above) and the database as a whole contains predominately low-intensity clips.

**Table 1:** Descriptive statistics (mean, standard deviation, and 95% confidence intervals) for intensity, valence, verbal cues, and sound quality of selected clips in Study 1

	Intensity	Valence	Verbal cues	Sound quality
Low intensity				
Posed	1.59 (0.28)	− 0.71 (0.70)	0.73 (1.00)	2.75 (0.60)
	[1.46, 1.72]	[− 1.03, − 0.38]	[0.26, 1.20]	[2.47, 3.03]
Spontaneous	1.48 (0.26)	− 0.31 (0.78)	0.65 (0.85)	2.95 (0.56)
	[1.36, 1.61]	[− 0.67, − 0.06]	[0.25, 1.05]	[2.69, 3.21]
Medium intensity				
Posed	2.38 (0.23)	− 0.67 (0.85)	0.46 (0.72)	2.87 (0.45)
	[2.27, 2.48]	[− 1.06, − 0.27]	[0.12, 0.79]	[2.66, 3.08]
Spontaneous	2.44 (0.25)	− 0.56 (1.11)	1.29 (0.89)	2.76 (0.56)
	[2.32, 2.55]	[− 1.08, − 0.04]	[0.87, 1.71]	[2.50, 3.03]
High intensity				
Posed	3.32 (0.22)	− 1.15 (1.28)	0.48 (0.45)	3.31 (0.27)
	[3.21, 3.44]	[− 1.81, − 0.49]	[0.25, 0.71]	[3.18, 3.45]
Spontaneous	3.36 (0.22)	− 1.63 (0.61)	1.52 (1.06)	2.50 (0.44)
	[3.26, 3.46]	[− 1.91, − 1.35]	[1.03, 2.02]	[2.29, 2.71]
Grand mean	2.40 (0.77)	− 0.83 (0.99)	0.87 (0.94)	2.85 (0.54)
	[2.26, 2.55]	[− 1.01, − 0.65]	[0.69, 1.04]	[2.75, 2.94]

#### Participants and Procedure

Thirty-two college students (16 female, ages = 22–42 years, *M* = 27.09) participated in the study. Their anonymous and voluntary participation was compensated with either course credits or cinema vouchers. Self-rated ability to understand the featured languages on a scale from 0 (*not at all*) to 4 (*very well*) was very high for both Swedish (*M* = 4.00, *SD* = 0.00) and English (*M* = 3.81, *SD* = 0.47), but considerably lower for French (*M* = 1.22, *SD* = 1.07) and German (*M* = 1.06, *SD* = 1.01). None of the participants reported a hearing problem. They received the following instructions:

You will soon hear a number of voice recordings containing women and men speaking in different languages. As you will hear, the speakers express various emotions. For each recording, your task is to judge if the speaker is experiencing a genuine (or “real”) emotion or if he or she is only pretending to experience the emotion. One might deliberately try to sound, for example, happy, angry, or sad, even though one is not actually feeling these emotions; or one might truly experience an emotion, which is spontaneously revealed through the voice. You make your judgments on a scale ranging from 0 (*not a genuine emotion at all*) to 4 (*a completely genuine emotion*). If you think it sounds as if the speaker is “moved” for real, you should choose a 4 on the scale. If you instead think it sounds as if the speaker is not experiencing a “real” emotion, then you should choose a 0 on the scale. If you think it sounds like a mixture of genuine and deliberately posed emotion, you should choose a 2 on the scale. The principle is always the same: The more you perceive the speaker to sound genuinely emotional, the higher the value you should choose. Note that you should not judge the strength or intensity of the expressed emotion, but only if the expression is genuine or not. Try to base your judgments on how the voice sounds, rather than the words that are spoken. Your focus should be on the tone of voice, not the verbal content.

Experiments were conducted individually, using the *Media Lab* software (Empirisoft, New York, USA) for stimulus presentation and response collection. Participants listened to the stimuli through a set of loudspeakers (*Dali Ikon 6 MK2*), with sound level kept constant across listeners. They were allowed to listen to each clip as many times as required to reach a decision. Stimulus order was randomized for each participant. Background questions were administered after the rating task. The length of a session was approximately 30 min.

### Results and Discussion

The consistency across raters was high, as indicated by an intra-class correlation (ICC) of .91 (two-way random model, average measures), *F* (116, 3596) = 10.74, *p* < .001. For each participant, we calculated the average ratings separately for spontaneous and posed clips with low, medium, and high emotion intensity. These values were entered into a two-way Analysis of Variance (ANOVA), within-subjects, with *stimulus type* (two levels) and *emotion intensity* (three levels) as factors. It is debatable whether the unit of analysis should be the judge or the target, but we chose to use a within-subjects design with judge as the unit of analysis because this was more statistically powerful than a between-subjects design with target as the unit.

Results revealed a significant main effect for *stimulus type*, *F*(1, 31) = 109.88, *p* < .001, partial η^2^ = 0.78. On average, spontaneous clips were rated as more genuine (*M* = 3.45, *SD* = 0.37) than posed clips (*M* = 2.66, *SD* = 0.43). In addition, there was a significant main effect of *emotion intensity*, *F*(2, 62) = 5.36, *p* = .007, partial η^2^ = 0.15. Specifically, clips with high intensity were generally rated as more genuinely emotional (*M* = 3.27, *SD* = 0.62), than clips with either medium (*M* = 2.96, *SD* = 0.39, *t*_31_ = 3.87, *p* < .001, *d* = 0.61) or low (*M* = 2.99, *SD* = 0.33, *t*_31_ = 2.22, *p* = .034, *d* = 0.59) intensity.

**Fig. 2:**
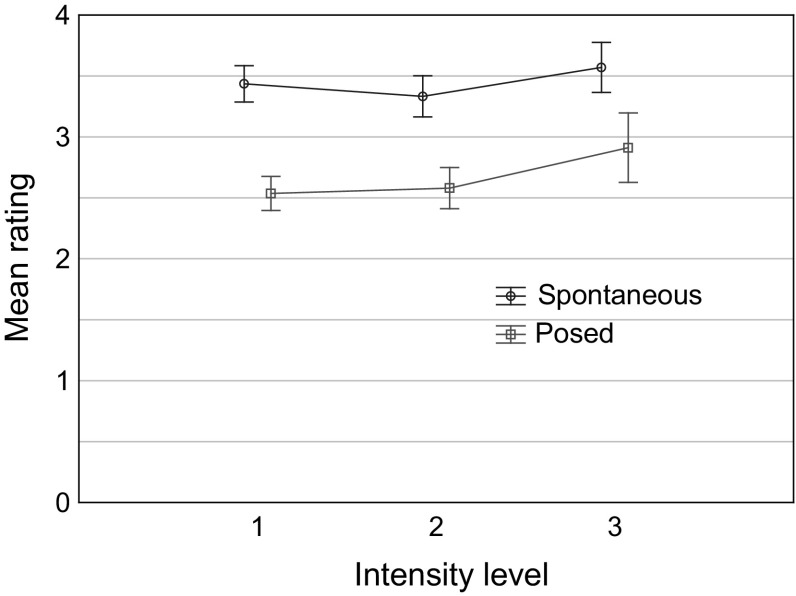
Means and 95% confidence intervals of listeners’ ratings (0–4) of the extent to which it sounds as if the speaker is experiencing a genuine emotion, for spontaneous and posed clips, respectively, as a function of emotion intensity level in Study 1

These main effects were qualified by a small but significant interaction, *F*(2, 62) = 3.47, *p* = .037, partial η^2^ = 0.10: see Fig. 2. Although spontaneous clips were consistently rated as more genuinely emotional than posed clips, the difference was smaller for high-intensity than for medium- and low-intensity clips.

The rated clips featured both familiar (Swedish) and unfamiliar languages. A follow-up analysis indicated that the main effects of *stimulus type*, *F*(1, 31) = 92.93, *p* < .001, partial η^2^ = 0.75, and *emotion intensity*, *F*(2, 62) = 3.96, *p* < .024, partial η^2^ = 0.11, remained significant when we excluded Swedish clips (*N* = 14), but the interaction was no longer significant, *F*(2, 62) = 1.47, *p* = .239, partial η^2^ = 0.05.

To further rule out confounding factors, we computed Pearson correlations between the listeners’ mean ratings of the 117 voice clips in the present study, and the mean ratings of the same clips in the pilot study. There were no significant correlations involving valence, *r*(115) = − .03, *p* = .786, verbal cues, *r*(115) = .09, *p* = .333, or sound quality, *r*(115) = − .06, *p* = .522, which renders it unlikely that these factors could account for the obtained difference between spontaneous and posed clips in this study. However, there was a tendency involving emotion intensity, *r*(115) = .18, *p* = .055, in line with the significant although small effect of emotion intensity discussed above.

## Study 2

### Introduction

Study 1 showed that spontaneous vocal expressions were perceived as more genuinely emotional than posed expressions, even after controlling for differences in emotion intensity, and that the difference was not due to confounding factors such as emotional valence, verbal cues to emotion, or a difference in sound quality. These findings suggest that the two sample types differ in some other way that helps the listener distinguish between them. One possible explanation is that they differ concerning their acoustic characteristics (Audibert et al. 2010; Jürgens et al. 2011), for instance by showing distinct acoustic patterns for the same emotion.

In Study 2, we analyzed the acoustic characteristics of the vocal expressions included in the novel database. To test for differences between stimulus types, we conducted an ANOVA-type analysis for each acoustic cue (see below) with emotion (e.g., sadness) and stimulus type (spontaneous vs. posed) as factors. Such a design could potentially reveal main effects of both emotion and stimulus type. However, the key effect when it comes to establishing a difference in cue patterns is the interaction between the two factors, which might reveal that the effect of emotion category is different depending on the stimulus type. Based on the results from Study 1, we expected to find at least *some* significant interactions. In addition to testing interactions, we aimed to investigate whether spontaneous vocal expressions would show emotion-specific patterns of cues, similarly to what has been shown for posed expressions (for an overview, see Juslin and Laukka 2003). Based on Spencer’s (1857) law that “feeling is a stimulus to muscular action” (p. 400; see Scherer 1986; Juslin and Laukka 2003), we hypothesized that spontaneous voice clips would indeed show emotion-specific patterns.

### Method

#### Acoustic Analysis

All 1877 clips from the database were acoustically analyzed for the purposes of the present study. The analyses were conducted by means of the openSMILE software (Eyben et al. 2013) to extract the voice parameters featured in the Geneva Minimalistic Acoustic Parameter Set (GeMAPS; for an overview, see Eyben et al. 2016).

The GeMAPS was recently proposed as a standardized “baseline-set” of acoustic cues relevant to vocal emotion expression, and features cues related to frequency, energy, spectral balance, and temporal aspects of the voice. The cues were selected by an international panel of experts, based on their potential to reflect physiological changes in voice production (e.g., Sundberg et al. 2011), the frequency and success with which they have been used in previous studies (e.g., Juslin and Laukka 2003), and their theoretical significance (e.g., Scherer 1986).

We used a pre-release version of the extended GeMAPS, containing 88 acoustic cues. Testing all 88 cues, however, would amount to a statistical “fishing expedition”. A principal components analysis (varimax normalized rotation and casewise deletion of missing values) was thus performed to reduce the number of cues included in subsequent statistical analysis. Outliers (values 3 SD above or below the mean) were excluded before data analysis in order to control for the occurrence of errors in the automatic extraction of cues (e.g., as caused by poor recording quality). The number of factors to retain was assessed using parallel analysis, as implemented in the *paran* package in R (Dinno 2009), and revealed a 13-factor solution. Based on the PCA results, we chose the cues with the highest loadings or interpretability for each factor. However, for two of the factors, there were no cues with loadings above .70, so we did not choose any cue to represent these factors. In addition to the 11 cues chosen based on the PCA results, we also featured two cues proposed based on prior research: speech rate (e.g., Barrett and Paus 2002) and jitter (e.g., Bachorowski and Owren 1995). The selected cues (*N* = 13) and their factor loadings are shown in Table 2.

**Table 2:** Summary of selected acoustic cues in Study 2

Feature type	Description	Factor loading
*Frequency cues*		
F0M	Mean fundamental frequency (F0) on a semitone frequency scale	Factor 2: 0.94
F0PercRange	Range of the 20th to the 80th percentile of F0	Factor 6: 0.92
F0SlopeRise	Mean slope of signal parts with rising F0	Factor 8: 0.89
F0SlopeFall	Mean slope of signal parts with falling F0	Factor 9: 0.84
F1 M	Mean frequency of the first formant (F1)	Factor 11: 0.75
F1Bandwidth	Mean bandwidth of the first formant (F1)	Factor 3: − 0.86
Jitter	Average deviation of individual consecutive F0 period lengths	(Factor 6: 0.64)
*Energy cues*		
IntPercRange	Range of the 20th to the 80th percentile of voice intensity	Factor 5: 0.90
*Spectral balance cues*		
Alpha Ratio	Ratio of the summed energy from 50 to 1000 Hz and 1000–5000 Hz	Factor 4: 0.73
H1-A3	Ratio of energy of the first F0 harmonic and the highest harmonic in the third formant range	Factor 13: − 0.71
*Temporal cues*		
VoicedSegPerSec	The number of continuous voiced regions per second (pseudo syllable rate)	(Factor 7: 0.61)
VoicedSegM	Mean length of continuously voiced regions	Factor 7: − 0.86
UnvoicedSegM	Mean length of unvoiced regions (approximating pause duration)	Factor 1: − 0.88

For a more comprehensive description of the acoustic cues, including algorithms used, see Eyben et al. (2013) and Eyben et al. (2016)

Acoustic cues were normalized, using z-transformation, prior to inclusion in statistical analyses. The normalization was performed separately for voice clips from female and male speakers to control for gender-related differences in voice characteristics and speech prosody.

#### Stimulus Material

We used the original emotion annotations from the datasets (Appendix 1) along with the emotion intensity ratings from the pilot study to prepare a set of spontaneous and posed voice clips that were matched concerning both emotion category and intensity. Information about the emotion expressed in each voice clip was readily available for all posed data sets, whereas only a few of the spontaneous data sets featured annotations of emotion categories (Gnjatovic and Rösner 2010; Juslin and Laukka 2017; Kehrein 2002; Scherer 2013; With and Kaiser 2011). However, we could use judgment data from the forced-choice experiment in Study 3 (described below) to include a couple of voice clips from spontaneous datasets that lacked category annotation. The analysis focused on the most frequently occurring emotion categories in the database: anger, fear, happiness, and sadness.

Appendix 3 shows the distribution of the selected clips across datasets and conditions. All available voice clips from *emotion* x *intensity* x *stimulus type* cells with frequency equal to or above 10 were included in the statistical analysis. (Stimuli with high intensity were not available in sufficient numbers to allow for a comparison of cue values across emotions.) In total, we were able to include 428 voice clips (spontaneous, *N* = 211; posed, *N* = 217) in the statistical analysis.

### Results and Discussion

The number of stimuli available for each condition varied a lot, as seen in Appendix 3. Because assumptions of normality and homoscedasticity were not met, we analyzed the data using robust ANOVA-type analyses instead of traditional analysis of variance (e.g., Wilcox 2012). Brunner et al. (1997) proposed a heteroscedastic rank-based permutation test using the *F* distribution, which may be calculated by means of the *bdm.2way* test in the *asbio* R-package (Aho 2015). For each of the chosen acoustic cues, we conducted between-groups ANOVA-type analyses with *stimulus type* and *emotion* as factors. Separate analyses were conducted for voice clips with low and medium emotion intensity, because the number of emotion categories differed across intensity levels.

The results of the robust ANOVA-type analyses are shown in Table 3.^1^ We also present the relative effects (Q; reflecting how the groups compare to each other, based on the average ranks) and descriptive statistics (*M*, *SD*) for each cue and condition in Tables 4 and 5. As may be seen, significant main effects of *emotion* were found for eight and five (out of 13) cues for low and medium intensity clips, respectively, showing that several cues varied as a function of emotion. The trends for the emotion effects are shown in the rightmost column of Table 3. We conducted post hoc comparisons, in the form of robust rank-based, Tukey-type nonparametric contrasts, using the *nparcomp* R-package (Konietschke et al. 2015). Results indicated, for instance, that happy voice clips featured higher pitch level (F0M) than sad clips, and that angry clips featured a higher speech rate (VoicedSegPerSec) than sad clips. The main effect of *stimulus type* was similarly significant for several cues, which shows that posed and spontaneous clips differed overall concerning the mean level of these cues (for details, see Table 3). As already discussed, however, differences in overall levels might occur even within the same stimulus type (e.g., posed clips). Therefore they do not constitute strong evidence of a difference between spontaneous and posed expressions.

**Table 3:** Robust analysis of acoustic cue variability in low and medium intensity posed and spontaneous vocal expressions in Study 2

Acoustic cue	Emotion		Stimulus type		Interaction		df1^a, b^	df2	Trends for the main effects of stimulus type and emotion
	F*	*p*	F*	*p*	F*	*p*			
*Low emotion intensity*									
F0 M	**9.47**	**.001**	**6.02**	**.016**	1.86	.149	2.65	77.99	P > S; Hap, Fea, Ang > Sad
F0PercRange	**7.28**	**.001**	1.35	.999	**3.60**	**.021**	2.70	79.11	Hap, Ang > Sad
F0SlopeRise	1.90	.139	0.34	.562	0.47	.688	2.80	84.41	na
F0SlopeFall	1.55	.217	1.38	.245	1.52	.223	2.52	54.61	na
F1 M	2.50	.077	0.08	.775	1.59	.206	2.50	71.43	na
F1 Bandwidth	**6.47**	**.003**	0.56	.457	0.46	.653	2.17	41.11	Sad > Ang, Hap, Fea
Jitter	2.01	.123	0.38	.539	1.27	.290	2.77	86.02	na
IntPercRange	**5.09**	**.005**	**9.39**	**.003**	**4.27**	**.011**	2.62	66.87	P > S; Hap > Sad
Alpha Ratio	**2.79**	**.048**	2.74	.101	1.51	.219	2.79	100.28	Ang > Sad
H1-A3	**5.78**	**.002**	**4.58**	**.036**	0.62	.592	2.73	72.95	P > S; Sad > Ang, Hap
VoicedSegPerSec	**3.20**	**.035**	0.39	.533	**3.54**	**.025**	2.62	59.75	Ang > Sad, Fea
VoicedSegM	2.67	.059	**11.87**	**.001**	**3.82**	**.016**	2.73	70.97	P > S; na
UnvoicedSegM	**6.01**	**.002**	0.11	.736	**2.94**	**.048**	2.55	58.08	Sad > Ang

Acoustic cue	Emotion		Stimulus type		Interaction		df1^a, b^	df2	Trends for the main effects of stimulus type and emotion
	F*	*p*	F*	*p*	F*	*p*			
*Medium emotion intensity*									
F0 M	**13.28**	**.001**	**5.46**	**.023**	1.34	.271	1.88	50.96	P > S; Hap, Ang > Sad
F0PercRange	0.09	.905	0.07	.788	2.44	.101	1.84	55.04	na
F0SlopeRise	1.67	.196	3.52	.064	**6.14**	**.004**	1.93	73.33	na
F0SlopeFall	0.24	.769	**15.71**	**.001**	2.48	.096	1.84	64.42	S > P; na
F1 M	**10.19**	**.001**	**5.18**	**.025**	1.87	.161	1.96	86.22	P > S; Ang, Hap > Sad
F1 Bandwidth	1.17	.316	0.74	.392	2.65	.083	1.86	61.69	na
Jitter	0.69	.494	**5.01**	**.029**	**7.59**	**.002**	1.84	54.75	S > P; na
IntPercRange	**8.64**	**.001**	0.29	.589	**5.51**	**.006**	1.94	80.69	Ang > Sad
Alpha Ratio	0.89	.411	0.01	.922	1.64	.201	1.94	73.41	na
H1-A3	0.94	.390	0.35	.554	0.47	.618	1.91	84.69	na
VoicedSegPerSec	**5.89**	**.005**	1.33	.253	0.52	.582	1.84	69.18	Ang > Sad
VoicedSegM	0.09	.907	0.93	.338	0.57	.561	1.95	71.06	na
UnvoicedSegM	**7.09**	**.002**	**4.78**	**.032**	1.52	.227	1.77	64.35	P > S; Sad > Hap

Significant effects from ANOVA-type analyses are marked in bold. Multiple comparisons assessing main trends for emotion were conducted using robust rank-based Tukey-type nonparametric contrasts (*p*s < .05)

F* = the ANOVA-type statistic, P = posed clips, S = spontaneous clips, Ang = anger, Fea = fear, Hap = happiness, Sad = sadness. Abbreviations of acoustic cues are explained in Table 2

^a^df1 = 1 for the main effect of stimulus type

^b^Reported df-values are Box-corrected, see Brunner et al. (1997)

**Table 4:** Descriptive statistics for acoustic cues in low intensity voice clips of Study 2

Acoustic cue	Statistic	Anger		Fear		Happiness		Sadness	
		P	S	P	S	P	S	P	S
F0M	*Q*	0.49	0.51	0.61	0.52	0.70	0.48	0.38	0.29
	*M*	− 0.30	− 0.24	0.11	− 0.31	0.36	− 0.38	− 0.68	− 0.99
	*SD*	0.79	0.82	1.01	1.07	0.65	0.82	0.92	0.80
F0PercRange	*Q*	0.58	0.58	0.44	0.55	**0.66**	**0.46**	0.31	0.40
	*M*	− 0.09	− 0.01	− 0.42	− 0.06	0.35	− 0.37	− 0.78	− 0.40
	*SD*	0.64	0.92	0.79	1.25	0.95	0.98	0.69	1.08
F0SlopeRise	*Q*	0.55	0.54	0.55	0.52	0.46	0.55	0.40	0.44
	*M*	0.19	0.04	− 0.06	0.00	− 0.07	0.04	− 0.30	− 0.28
	*SD*	1.11	0.94	0.91	1.06	0.90	0.89	1.03	0.86
F0SlopeFall	*Q*	0.51	0.59	0.45	0.57	0.56	0.45	0.37	0.47
	*M*	− 0.09	0.29	− 0.23	0.01	0.06	− 0.41	− 0.51	− 0.10
	*SD*	0.94	1.10	0.86	1.04	0.81	1.18	0.84	0.96
F1M	*Q*	0.44	0.55	0.45	0.36	0.51	0.59	0.50	0.44
	*M*	− 0.35	− 0.01	− 0.36	− 0.65	− 0.16	− 0.02	− 0.28	− 0.41
	*SD*	0.82	1.03	0.72	0.89	0.92	0.91	0.57	0.70
F1 Bandwidth	*Q*	0.44	0.51	0.40	0.35	0.43	0.49	0.60	0.65
	*M*	− 0.20	0.10	− 0.31	− 0.51	− 0.20	0.00	0.30	0.64
	*SD*	0.55	1.09	0.75	1.19	0.92	1.49	0.90	0.99
Jitter	*Q*	0.56	0.53	0.50	0.53	0.57	0.52	0.35	0.50
	*M*	− 0.02	0.02	− 0.15	0.06	0.20	0.01	− 0.52	− 0.13
	*SD*	0.83	0.99	0.93	1.24	1.01	1.06	0.96	1.04
IntPercRange	*Q*	0.43	0.52	**0.51**	**0.22**	**0.68**	**0.48**	0.49	0.41
	*M*	− 0.38	− 0.17	− 0.14	− 0.92	0.47	− 0.19	− 0.36	− 0.54
	*SD*	0.97	0.93	1.03	0.86	1.16	1.15	0.55	0.69
Alpha Ratio	*Q*	0.53	0.50	0.54	0.66	0.42	0.59	0.45	0.45
	*M*	− 0.11	− 0.08	− 0.23	0.39	− 0.56	0.26	− 0.57	− 0.24
	*SD*	0.91	0.88	1.14	1.09	1.27	0.81	0.95	0.69
H1-A3	*Q*	0.52	0.42	0.51	0.51	0.45	0.35	0.70	0.55
	*M*	0.36	0.08	0.32	0.23	0.06	− 0.35	0.90	0.44
	*SD*	0.86	0.88	0.90	0.95	0.87	1.03	0.93	0.90
VoicedSegPerSec	*Q*	**0.43**	**0.63**	0.43	0.30	0.46	0.56	0.48	0.41
	*M*	− 0.29	0.29	− 0.36	− 0.82	− 0.23	0.03	− 0.22	− 0.34
	*SD*	0.99	0.88	0.80	0.86	0.87	1.06	0.92	1.00
VoicedSegM	*Q*	0.57	0.46	**0.55**	**0.22**	**0.62**	**0.45**	0.47	0.52
	*M*	0.02	− 0.19	− 0.14	− 0.92	0.37	− 0.29	− 0.24	0.02
	*SD*	0.90	0.79	0.74	1.05	1.00	1.03	1.10	0.99
UnvoicedSegM	*Q*	0.53	0.37	0.54	0.71	0.46	0.45	0.59	0.64
	*M*	0.13	− 0.06	0.30	1.05	0.19	0.15	0.60	0.60
	*SD*	0.87	0.94	0.93	1.48	1.05	1.28	0.87	1.23

Q = relative effects, P = posed clips, S = spontaneous clips. Significant differences in values between posed and spontaneous clips (posthoc pairwise Bonferroni corrected Brunner Munzel tests, *p* < .05) are marked in bold type. Cue abbreviations are explained in Table 2

**Table 5:** Descriptive statistics for acoustic cues in medium intensity voice clips of Study 2

Acoustic cue	Statistic	Anger		Happiness		Sadness	
		P	S	P	S	P	S
F0M	*Q*	0.54	0.54	0.65	0.48	0.37	0.20
	*M*	0.65	0.98	0.06	0.67	0.40	− 0.46
	*SD*	0.90	0.81	1.23	0.69	0.80	0.68
F0PercRange	*Q*	0.55	0.44	0.54	0.45	0.39	0.55
	*M*	0.57	0.49	0.13	0.18	0.23	0.51
	*SD*	1.01	0.99	1.47	0.81	0.74	1.00
F0SlopeRise	*Q*	0.57	0.48	**0.32**	**0.63**	0.56	0.60
	*M*	0.29	− 0.44	0.23	0.12	0.41	0.53
	*SD*	1.24	0.72	1.10	1.08	1.06	1.01
F0SlopeFall	*Q*	0.45	0.57	0.44	0.56	0.29	0.65
	*M*	0.06	0.02	− 0.61	0.32	0.47	0.47
	*SD*	0.72	1.19	0.84	0.93	1.06	0.90
F1M	*Q*	0.56	0.57	0.59	0.40	0.39	0.26
	*M*	0.64	0.66	− 0.09	0.68	0.05	− 0.52
	*SD*	1.20	0.94	0.80	1.10	1.15	0.80
F1Bandwidth	*Q*	0.46	0.58	0.51	0.36	0.55	0.46
	*M*	− 0.15	− 0.06	0.09	0.18	− 0.55	− 0.17
	*SD*	0.81	1.08	0.69	1.09	0.92	0.62
Jitter	*Q*	**0.55**	**0.41**	0.43	0.56	**0.37**	**0.73**
	*M*	0.33	− 0.04	− 0.25	− 0.15	0.48	0.83
	*SD*	0.87	1.00	1.24	0.76	1.01	1.02
IntPercRange	*Q*	0.59	0.53	0.54	0.43	**0.21**	**0.45**
	*M*	0.49	0.50	− 0.42	0.60	0.27	0.27
	*SD*	0.93	0.93	0.69	0.98	1.06	0.71
Alpha Ratio	*Q*	0.46	0.58	0.54	0.44	0.45	0.43
	*M*	0.07	0.53	− 0.04	0.64	0.29	0.20
	*SD*	1.15	0.83	1.14	0.69	0.65	0.74
H1-A3	*Q*	0.51	0.48	0.44	0.52	0.54	0.58
	*M*	− 0.39	− 0.68	− 0.37	− 0.55	− 0.38	− 0.19
	*SD*	0.91	0.93	0.77	0.94	0.77	0.94
VoicedSegPerSec	*Q*	0.54	0.54	0.52	0.55	0.31	0.43
	*M*	0.42	0.14	− 0.44	0.45	0.26	− 0.02
	*SD*	1.19	1.01	0.43	1.11	1.01	0.86
VoicedSegM	*Q*	0.48	0.49	0.51	0.52	0.44	0.57
	*M*	− 0.01	0.29	− 0.03	0.22	0.27	0.52
	*SD*	1.02	1.14	0.93	1.04	1.17	0.96
UnvoicedSegM	*Q*	0.50	0.45	0.46	0.42	0.76	0.54
	*M*	− 0.42	− 0.45	0.40	− 0.45	− 0.59	− 0.30
	*SD*	0.70	0.88	1.05	0.72	0.57	0.79

Q = relative effects, P = posed clips, S = spontaneous clips. Significant differences in values between posed and spontaneous clips (posthoc pairwise Bonferroni corrected Brunner Munzel tests, *p* < .05) are marked in bold type. Cue abbreviations are explained in Table 2

**Fig. 3:**
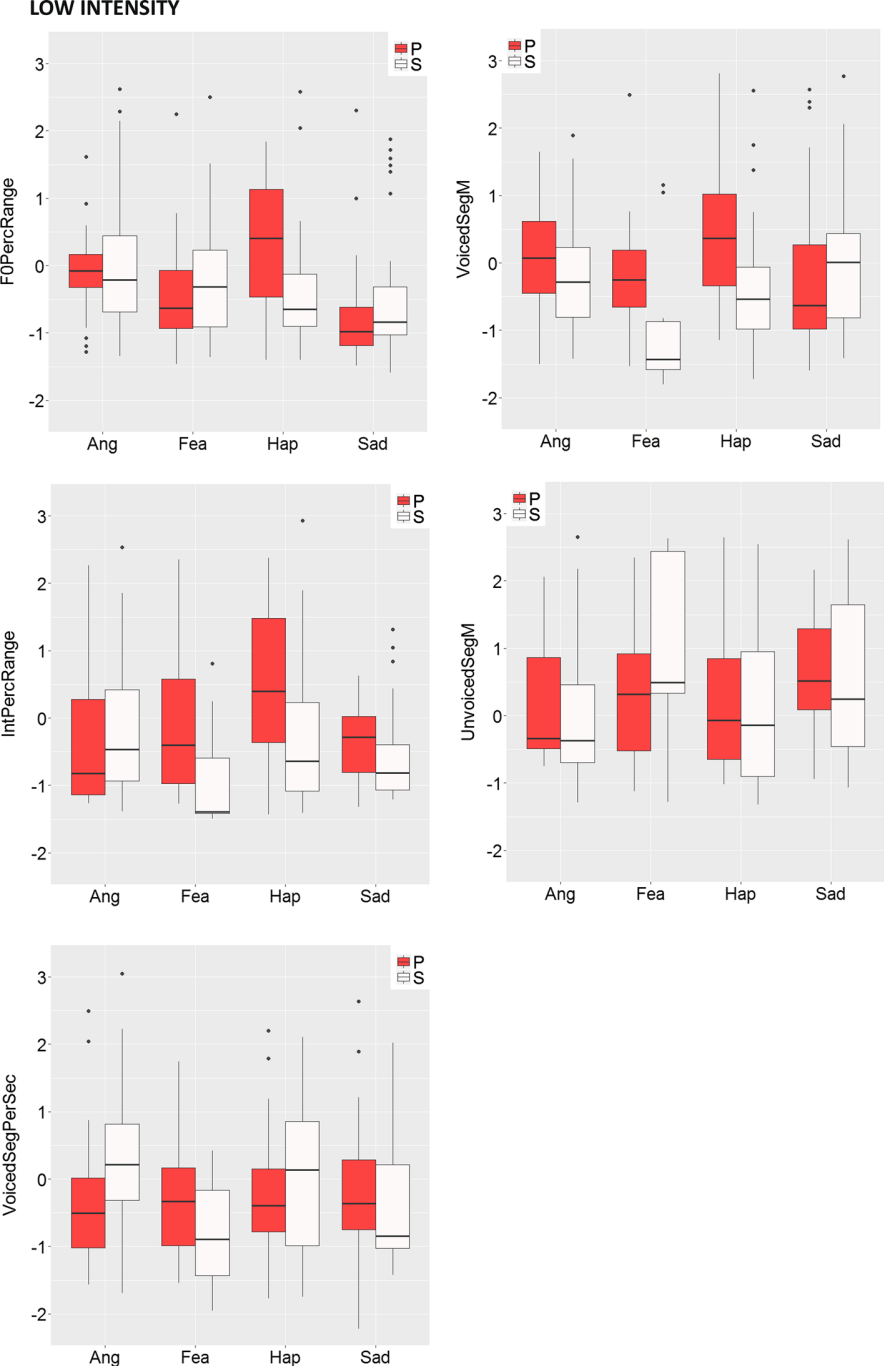

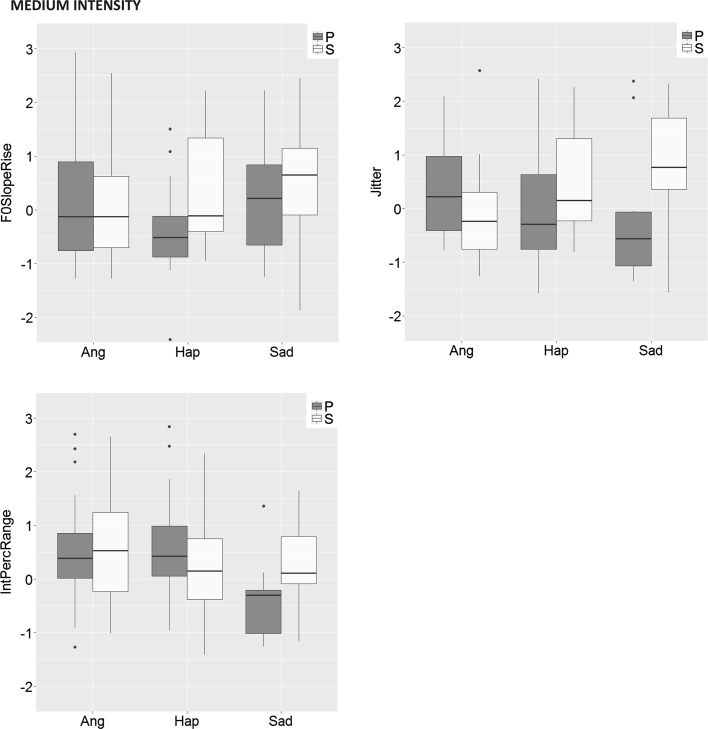
Box-and-whisker diagrams for all significant Stimulus-type x Emotion interactions in Study 2, for low and medium emotion intensity, respectively. P = posed clips, S = spontaneous clips, Ang = anger, Fea = fear, Hap = happiness, Sad = sadness. Values indicate z-scores. Cue abbreviations are explained in Table 2

The effect of main interest for the question whether emotions are expressed differently in spontaneous expressions as compared to posed expressions is the *stimulus type x emotion interaction.* It can be seen in Table 3 that for low-intensity stimuli, the interaction effect was significant for five out of 13 voice cues, and for medium-intensity stimuli for three cues. All significant interactions are displayed in Fig. 3.

The interactions were further explored using post hoc pairwise Brunner Munzel tests for independent groups (Konietschke et al. 2015).^2^ The following differences between posed and spontaneous clips remained significant (*p* < .05) after Bonferroni correction. For low-intensity clips, the results showed that pitch variability (F0PercRange) was larger for posed happy clips than for spontaneous happy clips (*W*_52.05_ = 3.05, *p* = .004, p-hat = 0.72); voice intensity range (IntPercRange) was wider for posed fear clips than for spontaneous fear clips (*W*_10.91_ = 3.07, *p* = .011, p-hat = 0.81); speech rate (VoicedSegPerSec) was higher for spontaneous angry clips than for posed angry clips (*W*_35.07_ = 3.11, *p* = .004, p-hat = 0.70); and voiced periods (Voiced SegM) were longer for posed happy clips than for spontaneous happy clips (*W*_45.20_ = 2.77, *p* = .008, p-hat = 0.70).

With respect to medium-intensity clips, spontaneous happy clips contained faster rising pitch movement (F0SlopeRise) than posed happy clips (*W*_17.69_ = 3.18, *p* = .005, p-hat = 0.77); posed angry clips featured more jitter than spontaneous angry clips (*W*_66.70_ = 2.49, *p* = .015, p-hat = 0.66); and, finally, spontaneous sad clips featured a wider intensity range (IntPercRange) than posed sad clips (*W*_23.94_ = 3.28, *p* = .003, p-hat = 0.79).

## Study 3

### Introduction

Studies 1 and 2 indicated that spontaneous and posed expressions differ to some extent, both perceptually and acoustically. Some scholars have suggested that such differences could reflect a far more serious problem: that spontaneous vocal expressions do not actually convey discrete categories of emotion (cf. Russell et al. 2003). However, based on Plutchik’s (1994) cone model of emotion (Introduction), one might hypothesize that previous studies of spontaneous expression have failed to obtain evidence of emotion-specific patterns of voice cues because they featured only low-intensity clips.

In Study 3, we examined this issue by means of a listening experiment featuring forced-choice judgments of all spontaneous voice clips from Study 1. We did not feature posed clips in this study because a previous meta-analysis based on 60 experiments has already provided clear evidence that posed clips convey discrete emotions to listeners (Juslin and Laukka 2003). Hence, even the sternest critics of the discrete emotions approach acknowledge that there is a moderate degree of emotion differentiation in posed clips, but they maintain that it is *because* they are posed clips (e.g., Russell et al. 2003). They argue that discrete emotions will not be evident in spontaneous clips. Still, considering that we observed emotion-specific patterns of voice cues in the spontaneous clips of medium emotion intensity in Study 2, we had reason to believe that listeners might also *perceive* discrete emotions in these same stimuli.

Our previous research has suggested that posed clips with high intensity involve more discrete and easily recognizable emotions than do posed clips with low intensity (e.g., Juslin and Laukka 2001). Based on these findings, and on Plutchik’s cone model, we predicted that there would be a dose–response relationship between intensity and discreteness in perceived emotions for spontaneous voice clips also. This tendency should be evident as better listener agreement for high-intensity clips than for low-intensity clips in forced-choice judgments of discrete emotions.

### Method

#### Participants and Procedure

Seventeen college students^3^ (nine females, ages = 24–74 years, *M* = 40.82) took part in the study. Their anonymous and voluntary participation was compensated with either course credits or cinema vouchers. Self-rated ability to understand the featured languages on a scale from 0 (*not at all*) to 4 (*very well*) was very high for both Swedish (*M* = 4.00, *SD* = 0.00) and English (*M* = 3.88, *SD* = 0.34), but considerably lower for French (*M* = 1.19, *SD* = 1.17) and German (*M* = 1.56, *SD* = 1.21). None of the participants reported a hearing problem.

The participants were required to rate all spontaneous voice clips (*N* = 60) from Study 1, in an emotion-recognition task using forced choice. Clips were presented in random order, and participants were asked to indicate the emotion conveyed by each utterance, by choosing one of the following emotion categories: *anger*-*irritation*, *fear*-*anxiety*, *sadness*-*melancholy*, *happiness*-*elation*, *disgust*-*contempt*, *surprise*-*astonishment*, *boredom*-*indifference*, and *calm*-*contentment*. The response options were based on the description of the featured spontaneous datasets (see Appendix 1) and on reviews of the most frequently occurring emotion categories in previous studies (see Juslin and Scherer 2005, Table 3.4). It has been argued that presenting participants with a limited number of response options may inflate decoding accuracy simply because participants are unable to choose other, potentially more applicable response options. Frank and Stennett (2001) demonstrated that this problem might be alleviated by introducing an additional response option that the participant may choose if none of the provided options seems appropriate. Hence, we also included the response option *other emotion*.

Participants listened to the stimuli through headphones, with sound level kept constant across listeners. They were allowed to listen to each clip as many times as required to reach a decision. Listening tests were conducted individually, using *Media Lab* software (Empirisoft, New York, USA). As in Study 2, background questions were administered after the judgment task. The length of a session was approximately 30 min.

### Results and Discussion

Emotion recognition studies typically calculate measures of decoding accuracy, such as percentage of correct responses, but in our case it is not possible to calculate a direct measure of accuracy because for many of the included clips, we do not know which emotions they are supposed to express. It is, therefore, not possible to calculate the Unbiased Hit Rate (Wagner 1993). However, the response alternative that was chosen by the majority of participants may be viewed as a post hoc criterion for correct response, in line with how many previous studies have operationalized accuracy in terms of rater agreement (Plutchik 1994).

The *mean dominant response*, defined as the percentage of judges that choose the most commonly chosen alternative, was 72% for the voice clips with high emotion intensity. This is six times higher than the level of chance performance expected in a forced choice task with nine response alternatives (i.e., 11%). This result clearly suggests that the high-intensity clips conveyed discrete emotions to listeners. As expected, moreover, the mean dominant response was lower for medium (58%) and low intensity (46%) clips.

Other measures tell essentially the same story. Thus, for example, if we look at *percent agreement* (the total number of times in which the raters agree, divided by the total number of classifications made), it was higher for high-intensity clips (59%) than for medium (42%) and low (28%) intensity clips. Similarly, *Fleiss’s Kappa* (a measure of interrater agreement across raters who assign a set of items to multiple categories) was higher for high-intensity clips (.43) than for the medium- (.33) and low-intensity (.17) clips. There are no universally agreed upon guidelines for interpreting Kappa values, though values exceeding .40 have been suggested to reflect “moderate” strength of agreement (Altman 1991). However, Kappa values decrease as the number of response options increases. Hence, it can be considered a conservative measure in the present experiment featuring nine categories.

Table 6 shows the distribution of responses across emotion categories, as a function of emotion intensity. As can be seen, typical “basic” emotions such as *anger*, *fear*, *sadness*, and *disgust* were most common amongst the high-intensity clips, whereas low-arousal emotions, such as *boredom* and *contentment*, were most frequent amongst the low-intensity clips. Note further that *happiness* was most common amongst the medium-intensity clips. Responses are most widely distributed across the emotion categories for the low-intensity clips. This can be interpreted as showing that these clips conveyed a large number of different emotions, but the low inter-rater agreement shown above suggests that a more parsimonious explanation is that low-intensity clips were more perceptually ambiguous in emotional meaning than other clips.

**Table 6:** Frequency distribution of perceived emotion categories in spontaneous vocal expressions with different levels of emotion intensity in Study 3

	Low	Medium	High
Anger	0.11	0.25	0.45
Fear	0.06	0.08	0.11
Sadness	0.10	0.15	0.24
Happiness	0.08	0.16	0.03
Disgust	0.05	0.06	0.11
Surprise	0.08	0.07	0.02
Boredom	0.16	0.08	0.01
Contentment	0.24	0.07	0.01
Other	0.14	0.08	0.03
Total	1.00	1.00	1.00

## General Discussion

The aim of this investigation was to compare spontaneous and posed vocal expression in order to examine whether they really are different. This issue was addressed in a series of experiments featuring samples from a novel and more representative database of voice clips than has been used in previous comparisons.

### Perceptual Differences

The results suggest that spontaneous and posed expressions *are* different – although not necessarily in the way commonly believed. In fact, a number of commonly held notions about possible differences were rejected (explained further below). In looking closer at the findings, we need to distinguish between two questions: (a) whether the currently available spontaneous and posed datasets tend to differ, and (b) whether spontaneous and posed expressions differ in a more *general* sense (i.e., apart from design artifacts or the effects of extraneous variables).

Regarding the first issue, the pilot study showed that the currently available databases of spontaneous and posed voice clips differ in several respects. On average, the posed clips had higher emotion intensity, conveyed more negative valence, had fewer verbal cues to emotion, and featured better recording quality. These effects were mostly “small”, in terms of Cohen’s (1988) guidelines for interpretation, but confirm the intuitions of researchers in the domain.

The observed differences reflect the typical design of studies using emotion portrayals: Actors are commonly instructed to portray strong emotions; to convey basic emotions, which feature more negative than positive categories; and to use a standardized and “neutral” verbal content. Moreover, portrayals are primarily recorded using high-quality equipment in a silent laboratory, as opposed to noisy field recordings of naturally occurring vocal expressions.

As noted previously, these differences could probably be eliminated or reduced simply in terms of the research design used. This is true also of the factor for which we observed the largest difference between the two sample types (*d* = .39); that is, emotion intensity. Note that the differences in emotion intensity between spontaneous and posed voice clips are not given by nature; they are a direct consequence of how the samples have been obtained. In principle, there is nothing to prevent researchers from recording a portrayal with low intensity (Juslin and Laukka 2001) or a spontaneous expression with high intensity (Juslin and Laukka 2017).

In recognition of this circumstance, we attempted in Study 1 to look beyond the current state of the datasets in order to resolve the second and arguably more important issue: Are the two types of sample different in a more general sense when controlling for various extraneous variables? Indeed, Study 1 showed that spontaneous expressions were generally rated as more genuinely emotional than posed expressions, even after controlling for differences in emotion intensity. This perceptual difference did not appear to be due to differences in valence, verbal cues, or sound quality, because none of these factors correlated with the extent to which a clip was rated as genuinely emotional. Furthermore, the raters showed a high level of consistency, ruling out that the observed trend was only spurious.

The present findings run counter to some previous suggestions in the literature. Thus, for instance, the notion that emotion intensity could account for the observed differences between spontaneous and posed vocal expression was not supported. Another common notion rejected by our results, is that posed expressions sound less authentic because they are more “aroused” and “stereotypical” than spontaneous expressions (cf. Cowie and Cornelius 2003; Jürgens et al. 2011). In fact, Study 1 indicated that, other things being equal, expressions with high intensity were generally rated as *more* “genuine”, than expressions with either medium or low intensity (though the effect was relatively small). The reasons for this result are not clear, but we could speculate that listeners consider it more difficult to “fake” highly intense vocal expressions of emotions in a convincing way, than it is to “fake” low- or medium- intensity expressions. This could explain the non-linear effect of emotion intensity (cf. Figure 2).

### Acoustic Differences

Study 2 revealed some further differences between spontaneous and posed expressions regarding acoustic characteristics, although the differences were relatively few, on the whole. The differences did not pertain only to the absolute level of cues, but also involved somewhat different patterns of cue values. These are relatively subtle acoustic differences that listeners might be able to detect. The findings suggest that the differences mainly involve measures of fundamental frequency (range, contour, jitter) and voice intensity, as proposed previously (cf. Audibert et al. 2010; Jürgens et al. 2011; Juslin and Laukka 2001), and also perhaps measures of speech rate. However, most voice cues showed similar tendencies across sample types, and the emotion trends were largely similar to those found in previous studies (see Tables 7 and 8 in Juslin and Laukka 2003).

Some authors have suggested that spontaneous expressions mainly convey “the general arousal level” and that “the still unanswered question is whether reliable patterns beyond this relationship can be established” (Russell et al. 2003, p. 340). However, we did find emotion-specific patterns of acoustic measures for spontaneous expressions in Study 2, like we did for posed expressions, as evidenced by a lack of *emotion x stimulus type* interactions for most of the cues. This finding is inconsistent with the view that only posed expressions have emotion-specific patterns. The data suggest that posed expressions are similar to–but not identical to–spontaneous expressions, similarly to what has been found in facial expression research (e.g., Ekman 1997). This conclusion receives some support by previous findings that the ability to decode spontaneous expression is positively although not perfectly correlated with the ability to decode posed expression (Rosenthal 1987).

### Discrete Emotions

The results from Study 3 clearly suggest that spontaneous expressions with high emotion intensity conveyed discrete emotions (e.g., sadness, happiness, anger) to listeners. Indeed, in a forced-choice listening test, 72% of the participants on average chose the response alternative that was most common to characterize the high intensity expressions. This level of agreement is at the very least similar to the accuracy estimates seen in reviews of studies based primarily on posed expressions (Scherer 1986), most of which involve high intensity. Moreover, Study 3 suggested that high-intensity clips were more discrete than low-intensity clips–as predicted by Plutchik’s (1994) cone model of emotion. This supports the hypothesis that lower levels of agreement in studies which used spontaneous expressions are primarily due to lower levels of emotion intensity in the clips used. This can be related to findings showing that high-intensity portrayals produce higher levels of decoding accuracy than do low-intensity portrayals (Juslin and Laukka 2001). It would appear that *both* spontaneous and posed vocal expressions involve more discrete and easily recognizable emotions as the intensity increases. Similar results have been found in studies of facial expression (Tassinary and Cacioppo 1992).

### Limitations of the Present Research

The above conclusions notwithstanding, there are a number of limitations in the present experiments that should be acknowledged. First, we only included voice clips that consisted of a single grammatical sentence. Strictly speaking, then, our conclusions must be limited to these conditions. Similarly, our database was limited to five European languages, for which we could obtain a sufficient collection of datasets. Because language could influence results in this domain (Scherer 2013), we should be wary of generalizing to other languages.

One further limitation, mentioned in the pilot study, is that we were not able to obtain all datasets that met our criteria for inclusion, which is illustrative of more general problems in the present field, such as copyright or privacy restrictions, which prevent sharing of audio recordings. It must be considered something of a failure that, after all recent efforts to create new emotion-in-speech databases, we experienced such difficulties in obtaining sufficiently large samples to systematically compare stimuli.

Yet, we featured what is arguably the most representative sample of voice clips in any comparison of posed and spontaneous expressions so far, which strengthens our conclusions. We also tried in all sorts of ways to make the comparisons as fair as possible, controlling for intensity, valence, verbal cues, and sound quality, and sampling voice clips in a randomized manner, to avoid selection bias. We would have preferred to control for individual emotions also, but this was not feasible, given the large disparity between the databases with regard to emotional content and annotations.

### Implications for Future Research

What are the implications of the present study for the use of posed vocal expressions in emotion research in general, and speech databases in particular? One clear implication is that researchers need to be cautious–it cannot simply be assumed that posed clips will be similar to spontaneous ones. Having said that, the differences do not appear to be many and there are high-quality portrayals that may be indistinguishable from spontaneous expressions for most lay listeners. This shows that portrayals could fulfill the requirements of emotion researchers as long as they go through a quality check (e.g., ensuring that they are indistinguishable from a spontaneous expression for listeners, and checking that voice-cue patterns are qualitatively the same as those for the corresponding spontaneous ones). The consequences of using posed instead of spontaneous clips could depend on the purpose of a study or a practical application (Scherer and Bänziger 2010). To be fair, many researchers using emotion portrayals seem well aware of the risks and discuss various means to ensure that the portrayals are adequate (Banse and Scherer 1996; Scherer et al. 1991)–including the use of emotion scenarios, mood-induction techniques, and listening tests to assess the “believability” of clips.

Notably, the use of professional actors does *not* seem to guarantee adequacy. One study found that listeners rated portrayals by professional actors as *less* similar to “real” expressions than portrayals by non-professionals (Krahmer and Swerts 2008). On the other hand, it has also been suggested that acting skills have become more “realistic” over time, and that the forensic nature of modern high-definition film places greater demands on “naturalistic detail” (Norman 2014). It seems that a key task for the future is to develop better means to verify the quality of emotion portrayals. Doing so requires that we have an adequate understanding of spontaneous expressions. The present investigation shows that we still have some way to go in that respect.

It has been questioned whether it is feasible to make a distinction between spontaneous and posed expressions, or “push” and “pull” effects (see Scherer and Bänziger 2010; Tatham and Morton 2004, p. 208). However, the present results in Study 1 and 2 strongly suggest that the distinction is meaningful: posed expressions were generally rated as less genuinely emotional and also tended to have different acoustic patterns. However, as argued by Banse and Scherer (1996), whereas posed voice clips may not be “natural enough”, spontaneous clips may not be “emotional enough”. This much was apparent in the present studies, which exposed a number of flaws in current datasets with spontaneous and supposedly emotional speech. For example, the obtained overall differences in emotion intensity between spontaneous and posed datasets reflect in no small part that some spontaneous clips lacked emotion altogether.

The noted difficulty in obtaining spontaneous expressions of strong emotions (Douglas-Cowie et al. 2003) has led to a puzzling choice of direction in the domain: Rather than trying harder to obtain emotional voice clips, researchers have suggested looking at milder affective states (Cowie and Cornelius 2003), as if convenience should determine the research focus. The problem with such an approach is highlighted by the present investigation. A focus merely on low-intensity clips may lead to conclusions which are incomplete or misleading. To illustrate, we found that spontaneous expressions really are different from posed expressions, but not in the simplistic sense that they are less “stereotypical” or less “discrete”. Instead, they appear to differ with regard to more subtle acoustic nuances, which listeners may be able to detect. The precise nature of the voice cues that reveal genuine emotion remains to be described in future studies that take vocal expressions of all intensities into consideration.

## Appendix 1

See Table 7.

**Table 7:** Summary of the 23 datasets included in the database

Database	Description of content	Language	Initial content	Number of selected files	Type
Berlin (Burkhardt et al. 2005)	Portrayals of 6 emotions (anger, boredom, disgust, fear, joy, sadness) by 10 actors. 10 standard content sentences (same for all emotions)	German	535 audio files	119 (randomly selected)	Acted (professional)
eNTERFACE’05 (Martin et al. 2006)	Portrayals of 6 emotions (anger, disgust, fear, happiness, sadness, surprise) by 42 actors. 5 standard content sentences (different for each emotion)	English (non-native speakers)	1293 video files	89 (randomly selected)	Acted (non-professional)
GEMEP (Bänziger et al. 2012)	Portrayals of 18 emotions (admiration, amusement, anxiety, cold anger, contempt, despair, disgust, elation, hot anger, interest, panic fear, pleasure, pride, relief, sadness, shame, surprise, tenderness) by 10 actors. Free speech content (different for each portrayal)	French	1463 video files	83 (randomly selected)	Acted (professional)
Hawk et al. (2009)	Portrayals of 9 emotions (anger, contempt, disgust, embarrassment, fear, joy, pride, sadness, surprise) by 8 actors. One standard content sentence (same for all emotions)	English	72 audio files	0 (no content with full sentences)	Acted (acting students)
Juslin and Laukka (2001)	Portrayals of 5 emotions (anger, disgust, fear, happiness, sadness; each with 2 levels of emotion intensity) by 8 actors. 2 standard content sentences per language (same for all emotions)	English, Swedish	160 audio files	25 (randomly selected)	Acted (professional)
SAVEE (Haq and Jackson 2009)	Portrayals of 6 emotions (anger, disgust, fear, happiness, sadness, surprise) by 4 actors. 15 standard content sentences per emotion (3 common for all emotions)	English	360 video files	90 (randomly selected)	Acted (non-professional)
SU Voices (Nordström and Laukka 2017)	Portrayals of 13 emotions (anger, contempt, disgust, fear, happiness, interest, lust, pride, relief, sadness, serenity, shame, tenderness; each with 2 levels of intensity) by 14 actors. One standard content sentence (same for all emotions)	Swedish	364 audio files	27 (randomly selected)	Acted (professional and non-professional)
VENEC (Laukka et al. 2010)	Portrayals of 18 emotions (affection, anger, amusement, contempt, disgust, distress, fear, guilt, happiness, interest, lust, negative surprise, positive surprise, pride, relief, sadness, serenity, shame; each with 3 levels of emotion intensity) by 20 actors. 2 standard content sentences (same for all emotions)	English	1020 audio files	102 (randomly selected)	Acted (professional)
Belfast Naturalistic (Douglas-Cowie et al. 2000)	Recordings of human interactions. Annotated for arousal and valence	English	22 video files	20 (selected based on observer ratings)	Spontaneous (interviews)
BINED (Sneddon et al. 2012)	Recordings of participants engaging in various emotion inducing tasks in laboratory settings. Annotated for arousal and valence. Only a subset of recordings contains speech	English	28 long video files	67 (manually extracted and selected based on observer ratings)	Spontaneous (emotion inducing laboratory tasks)
DIT IE (Cullen et al. 2008)	Recordings of participants engaging in a computer game task. Annotated for arousal and valence	English	160 segmented and pre-selected audio files	54 (selected based on observer ratings)	Spontaneous (emotion inducing laboratory tasks)
E-Wiz (Aubergé et al. 2004)	Recordings of participants engaging in a Wizard-of-Oz task. We used a pre-selected set of annotated stimuli, as described in Laukka et al. (2012)	French	36 segmented and pre-selected audio files	5 (selected based on observer ratings; most of the content consisted of single word utterances)	Spontaneous (emotion inducing laboratory tasks)
HUMAINE (Douglas-Cowie et al. 2007, 2011)	Recordings from various sources, including reality television shows, emotion inducing lab tasks, and human interactions. Some of the content is annotated for arousal and valence	English, French	47 long video files	55 (manually extracted and selected based on observer ratings)	Spontaneous (interviews, emotion inducing laboratory tasks, television shows)
Lego (Kehrein 2002)	Recordings of dialogues where participants cooperatively try to assemble an impossible Lego task. Annotated with regard to various affect labels	German	5 long audio files	118 (manually extracted and selected based on observer ratings)	Spontaneous (emotion inducing laboratory task)
Nimitek (Gnjatović and Rösner 2010)	Audio recordings of participants engaging in a Wizard-of-Oz task. Partly annotated with regard to various affect labels	German	10 long audio files	113 (selected based on observer ratings)	Spontaneous (emotion inducing laboratory tasks)
SEMAINE (McKeown et al. 2012)	Video recordings of human interactions. Annotated for arousal and valence	English	140 long video files	210 (manually extracted and selected based on observer ratings)	Spontaneous (interviews)
SSPNet Conflict (Kim et al. 2014)	Recordings from televised political debates. Annotated with regard to conflict level	French	1430 audio segments	162 (manually extracted and selected based on observer ratings)	Spontaneous (television shows)
TIVAC (Juslin and Laukka 2017)	Recordings of emotional speech from various sources available online	English, Swedish	84 segmented and pre-selected audio files	84 (selected based on observer ratings)	Spontaneous (documentaries, television shows, interviews etc.)
TNO Gaming (Truong et al. 2012)	Recordings of persons engaging in a computer game task. Annotated for self-report and observer ratings of arousal and valence	Dutch	2400 segmented audio files	49 (selected based on self-report and observer ratings)	Spontaneous (emotion inducing laboratory task)
Vera am Mittag (Grimm et al. 2008)	Recordings from a television talk show. Annotated for perceived arousal, valence and dominance levels	German	947 video files	53 (selected based on observer ratings)	Spontaneous (television shows)
With and Kaiser (2011)	Recordings from clinical interviews. Annotated with regard to perceived affect labels	French	202 segmented and pre-selected video files	77 (selected based on observer ratings)	Spontaneous (interviews)
Voice provider (Neiberg et al. 2006)	Recordings from call center human–computer interactions. We used a pre-selected set of annotated stimuli, as described in Laukka et al. (2011)	Swedish	200 segmented and pre-selected audio files	4 (selected based on observer ratings; most of the content consisted of single word utterances)	Spontaneous (call center data)
Emovox (Klasmeyer et al. 2000; Scherer 2013)	Recordings of participants engaging in acting tasks and various emotion inducing lab tasks. Annotated by emotional self-ratings	English, French, German	Nearly 14,000 segmented audio files	Acted: 108 (randomly selected)	Acted (non-professional)
				Spontaneous: 163 (based on self-reported emotion ratings)	Spontaneous (emotion inducing laboratory tasks)

## Appendix 2

See Table 8.

**Table 8:** Distribution of randomly selected voice clips in Study 1 across datasets

Database	Low intensity	Medium intensity	High intensity	Total
*Posed expressions*				
Berlin (Burkhardt et al. 2005)	1	4	5	10
Emovox (Klasmeyer et al. 2000; Scherer 2013)	3	1		4
eNTERFACE’05 (Martin et al. 2006)	4	3		7
GEMEP (Bänziger et al. 2012)	6	4	10	20
Juslin and Laukka (2001)	1	2	1	4
SAVEE (Haq and Jackson 2009)	3	6		9
SU Voices (Nordström and Laukka 2017)	1			1
VENEC (Laukka et al. 2010)	1		1	2
*Spontaneous expressions*				
Belfast Naturalistic (Douglas-Cowie et al. 2000)	2			2
DIT IE (Cullen et al. 2008)	1	1	1	3
Emovox (Scherer 2013)	1			1
HUMAINE (Douglas-Cowie et al. 2007, 2011)	1			1
Lego (Kehrein 2002)	2			2
Nimitek (Gnjatović and Rösner 2010)	1	3	1	5
SEMAINE (McKeown et al. 2012)	5	4		9
SSPNet Conflict (Kim et al. 2014)	5	2		7
TIVAC_E (Juslin and Laukka 2017)		1	10	11
TIVAC_S (Juslin and Laukka 2017)		5	6	11
Vera am Mittag (Grimm et al. 2008)		4	2	6
With and Kaiser (2011)	2			2

## Appendix 3

See Table 9.

**Table 9:** Distribution of voice clips included in the acoustic comparisons of Study 2 across the datasets

Database	Anger	Anger	Fear	Happiness	Happiness	Sadness	Sadness
	Low	Medium	Low	Low	Medium	Low	Medium
*Posed expressions*							
Total	25	34	32	36	33	44	13
Berlin (Burkhardt et al. 2005)		7		5	14	17	2
Emovox (Klasmeyer et al. 2000; Scherer 2013)	7	1	6	3	1	4	1
eNTERFACE’05 (Martin et al. 2006)	5	8	9	4	8	9	1
GEMEP (Bänziger et al. 2012)	4	4		2	4	3	3
Juslin and Laukka (2001)	2	3	4	2	2	2	1
SAVEE (Haq and Jackson 2009)	4	9	8	12	1	6	1
SU Voices (Nordström and Laukka 2017)	1	1	1	2		1	1
VENEC (Laukka et al. 2010)	2	1	4	6	3	2	3
*Spontaneous expressions*							
Total	73	39	11	25	14	28	21
Belfast Naturalistic (Douglas-Cowie, et al. 2000)			1				
DIT IE (Cullen et al. 2008)					1		
Emovox (Scherer 2013)	16			2		11	2
E-Wiz (Aubergé et al. 2004)				1			
Lego (Kehrein 2002)	22	1		5	2	4	
Nimitek (Gnjatović and Rösner 2010)	19	15	2	8		9	
SEMAINE (McKeown et al. 2012)		1			1	1	
SSPNet Conflict (Kim et al. 2014)	2	3					
TIVAC_E (Juslin and Laukka 2017)		7		1	5	2	10
TIVAC_S (Juslin and Laukka 2017)	8	7		3	3	1	9
Vera am Mittag (Grimm et al. 2008)		3					
Voice Provider (Neiberg et al. 2006)	2	2					
With and Kaiser (2011)	4		8	5	2		

## Footnotes

1 Notably, the R software does not provide measures of effect size for the main effects and interactions of its robust ANOVA-type analyses, and due to a difference in computation, regular effect indices cannot be used.

2
*W* denotes the Brunner Munzel test statistic with associated degrees of freedom, and p-hat is an estimate of the effect size in terms of the probability that a randomly taken score from one group will be greater or smaller than a randomly taken score from the other group (i.e., stochastic superiority; see Wilcox, 2012).

3 We acknowledge that this listener sample is smaller than in Study 1, which might influence the stability of our estimates. However, because we do not apply significance testing to the data, the risk of a Type II error is not an issue here.

## References

[CR1] Aho, K. (2015). *asbio: A collection of statistical tools for biologists.* R package version 1.2.5.

[CR2] Altman DG (1991). Practical statistics for medical research.

[CR3] Aubergé, V., Audibert, N., & Rilliard, A. (2004). E-Wiz: A trapper protocol for hunting the expressive speech corpora in lab. In M. T. Lino et al. (Eds.), *Proceedings of the fourth international conference on language resources and evaluation* (pp. 179–182). Paris: European Language Resources Association.

[CR4] Audibert N, Aubergé V, Rilliard A, Barbosa PA, Madureira S, Reis C (2008). How we are not all equally competent for discriminating acted from spontaneous expressive speech. Fourth conference on speech prosody.

[CR5] Audibert, N., Aubergé, V., & Rilliard, A. (2010). Prosodic correlates of acted vs. spontaneous discrimination of expressive speech: A pilot study. In *Proceedings of Speech Prosody 2010* (pp. 1–4).

[CR6] Bachorowski J-A (1999). Vocal expression and perception of emotion. Current Directions in Psychological Science.

[CR7] Bachorowski J-A, Owren MJ (1995). Vocal expression of emotion: Acoustic properties of speech are associated with emotional intensity and context. Psychological Science.

[CR8] Banse R, Scherer KR (1996). Acoustic profiles in vocal emotion expression. Journal of Personality and Social Psychology.

[CR9] Bänziger T, Mortillaro M, Scherer KR (2012). Introducing the geneva multimodal expression corpus for experimental research on emotion perception. Emotion.

[CR10] Barrett J, Paus T (2002). Affect-induced changes in speech production. Experimental Brain Research.

[CR11] Brunner E, Dette H, Munk A (1997). Box-type approximations in nonparametric factorial designs. Journal of the American Statistical Association.

[CR12] Buck R (2014). Emotion: A biosocial synthesis.

[CR13] Burkhardt, F., Paeschke, A., Rolfes, M., Sendlmeier, W. F., & Weiss, B. (2005). A database of German emotional speech. In *Proceedings of the 9th European conference on speech communication and technology, Interspeech 2005* (pp. 1517–1520). Lisbon: International Speech Communication Association.

[CR14] Caffi C, Janney RW (1994). Toward a pragmatics of emotive communication. Journal of Pragmatics.

[CR15] Carletta JC (2007). Unleashing the killer corpus: Experiences in creating the multi-everything AMI meeting corpus. Language Resources and Evaluation.

[CR16] Cohen J (1988). Statistical power analysis for the behavioral sciences.

[CR17] Cowie R, Cornelius R (2003). Describing the emotional states that are expressed in speech. Speech Communication.

[CR18] Cowie R, Douglas-Cowie E, Tsapatsoulis N, Votsis G, Kollias S, Fellenz W, Taylor JG (2001). Emotion recognition in human–computer interaction. IEEE Signal Processing Magazine.

[CR19] Cullen, C., Vaughan, B., Kousidis, S., & McAuley, J. (2008). Emotional speech corpus construction, annotation, and distribution. In L. Devillers, J.-C., Martin, R. Cowie, E. Douglas-Cowie, & A. Batliner (Eds.), *Proceedings of the LREC 2008 workshop on corpora for research on emotion and affect* (pp. 32–37). Marrakesh: ELRA.

[CR20] Davitz JR, Davitz JR (1964). Auditory correlates of vocal expression of emotional feeling. The communication of emotional meaning.

[CR21] Dinno A (2009). Exploring the sensitivity of Horn’s parallel analysis to the distributional form of simulated data. Multivariate Behavioral Research.

[CR22] Douglas-Cowie E, Campbell N, Cowie R, Roach P (2003). Emotional speech: Towards a new generation of data bases. Speech Communication.

[CR23] Douglas-Cowie, E., Cowie, R., & Romano, A. (1999). Changing emotional tone in dialogue and its prosodic correlates. In M. Swerts & J. Terken (Eds.), *Proceedings of the ESCA workshop on dialogue and prosody* (pp. 41–46). Eindhoven: Eindhoven University.

[CR24] Douglas-Cowie, E., Cowie, R., & Schröder, M. (2000). A new emotion database: Considerations, sources, and scope. In R. Cowie, E. Douglas-Cowie, & M. Schröder (Eds.), *Proceedings of the ISCA workshop on speech and emotion* (pp. 39–44). Belfast: International Speech Communication Association.

[CR25] Douglas-Cowie E, Cowie R, Sneddon I, Cox C, Lowry O, McRorie M, Karpouzis K, Paiva A, Prada R, Picard RW (2007). The HUMAINE database: Addressing the collection and annotation of naturalistic and induced emotional data. Affective computing and intelligent interaction, ACII 2007.

[CR26] Douglas-Cowie E, Cox C, Martin J-C, Devillers L, Cowie R, Sneddon I, McRorie M, Hönig F, Petta P, Pelachaud C, Cowie R (2011). The HUMAINE database. Emotion-oriented systems: The Humaine handbook.

[CR27] Ekman P (1997). Should we call it expression or communication?. Innovation.

[CR28] Ekman P (1973). Darwin and facial expression.

[CR29] Ekman P, Cordaro D (2011). What is meant by calling emotions basic?. Emotion Review.

[CR30] Ekman P, Friesen WV (1969). The repertoire of nonverbal behavior: Categories, origins, usage, and coding. Semiotica.

[CR31] El Ayadi M, Kamel MS, Karray F (2011). Survey on speech emotion recognition: Features, classification schemes, and databases. Pattern Recognition.

[CR32] Eyben F, Scherer KR, Schuller BW, Sundberg J, André E, Busso C, Devillers LY, Truong KP (2016). The Geneva minimalistic acoustic parameter set (GeMAPS) for voice research and affective computing. IEEE Transactions on Affective Computing.

[CR33] Eyben, F., Weninger, F., Gross, F., & Schuller, B. (2013). Recent developments in openSMILE, the Munich open-source multimedia feature extractor. In J. Alejandro et al. (Eds.), *Proceedings of ACM multimedia 2013* (pp. 835–838). Barcelona: Association for Computing Machinery.

[CR34] Frank MG, Juslin PN, Harrigan JA, Harrigan JA, Rosenthal R, Scherer KR (2005). Technical issues in recording nonverbal behavior. The new handbook of methods in nonverbal behavior research.

[CR35] Frank MG, Stennett J (2001). The forced-choice paradigm and the perception of facial expressions of emotion. Journal of Personality and Social Psychology.

[CR36] Frick RW (1985). Communicating emotion: The role of prosodic features. Psychological Bulletin.

[CR37] Fridlund A (1994). Human facial expression: An evolutionary view.

[CR38] Frommer, J., Michaelis, B., Rösner, D., Wendemuth, A., Friesen, R., Haase, M., et al. (2012). Towards emotion and affect detection in the multimodal LAST MINUTE corpus. In N. Calzolari et al. (Eds.), *Proceedings of the 8th international conference on language resources and evaluation, LREC 2012* (pp. 3064–3069). Istanbul: European Language Resources Association.

[CR39] Gnjatović M, Rösner D (2010). Inducing genuine emotions in simulated speech-based human–machine interaction: The NIMITEK corpus. IEEE Transactions on Affective Computing.

[CR40] Greasley P, Sherrard C, Waterman M (2000). Emotion in language and speech: Methodological issues in naturalistic settings. Language and Speech.

[CR41] Grimm, M., Kroschel, K., & Narayanan, S. (2008). The Vera am Mittag German audio-visual emotional speech database. In *Proceedings of the 2008 IEEE international conference on multimedia and expo, ICME 2008* (pp. 865–868). Piscataway, NJ: Institute of Electrical and Electronics Engineers.

[CR42] Hansen J. H. L., & Bou-Ghazale, S. E. (1997). Getting started with SUSAS: A speech under simulated and actual stress database. In *Proceedings of the 5th European conference on speech communication and technology, Eurospeech’97* (pp. 1743–1746). Rhodes: European Speech Communication Association.

[CR43] Haq, S., & Jackson, P. J. B. (2009). Speaker-dependent audio-visual emotion recognition. In B.-J. Theobald & R. Harvey (Eds.), *Proceedings of the international conference on auditory-visual speech processing, AVSP 2009* (pp. 53–58). Norwich: International Speech Communication Association.

[CR44] Hawk ST, van Kleef GA, Fischer AH, van der Schalk J (2009). ‘Worth a thousand words’: Absolute and relative decoding of nonlinguistic affect vocalizations. Emotion.

[CR45] Izard CE, Lewis M, Haviland JM (1993). Organizational and motivational functions of discrete emotions. Handbook of emotions.

[CR46] Jürgens R, Grass A, Drolet M, Fischer J (2015). Effect of acting experience on emotion expression and recognition in voice: Non-actors provide better stimuli than expected. Journal of Nonverbal Behavior.

[CR47] Jürgens R, Hammerschmidt K, Fischer J (2011). Authentic and play-acted vocal emotion expressions reveal acoustic differences. Frontiers in Psychology.

[CR48] Juslin PN, Altenmüller E, Schmidt S, Zimmerman E (2013). Vocal expression of affect: Promises and problems. Evolution of emotional communication.

[CR49] Juslin PN, Laukka P (2001). Impact of intended emotion intensity on cue utilization and decoding accuracy in vocal expression of emotion. Emotion.

[CR50] Juslin PN, Laukka P (2003). Communication of emotions in vocal expression and music performance: Different channels, same code?. Psychological Bulletin.

[CR51] Juslin, P. N., & Laukka, P. (2017). *The truly intense vocal affect collection (TIVAC): Presentation and validation.* Manuscript submitted for publication.

[CR52] Juslin PN, Scherer KR, Harrigan JA, Rosenthal R, Scherer KR (2005). Vocal expression of affect. The new handbook of methods in nonverbal behavior research.

[CR53] Kappas A, Hess U, Quasthoff UM (1995). Nonverbal aspects of oral communication. Aspects of oral communication.

[CR54] Kehrein, R. (2002). The prosody of authentic emotions. In B. Bel & I. Marlien (Eds.), *Proceedings of the speech prosody 2002 conference* (pp. 423–426). Aix-en-Provence: Université de Provence.

[CR55] Kim S, Valente F, Filippone M, Vinciarelli A (2014). Predicting continuous conflict perception with Bayesian Gaussian processes. IEEE Transactions on Affective Computing.

[CR56] Klasmeyer, G., Johnstone, T., Bänziger, T., Sappok, C., & Scherer, K. R. (2000). Emotional voice variability in speaker verification. In R. Cowie, E. Douglas-Cowie, & M. Schröder (Eds.), *Proceedings of the ISCA workshop on speech and emotion* (pp. 212–217). Belfast: International Speech Communication Association.

[CR57] Konietschke F, Placzek M, Schaarschmidt F, Hothorn LA (2015). nparcomp: An R software package for nonparametric multiple comparisons and simultaneous confidence intervals. Journal of Statistical Software.

[CR58] Krahmer, E., & Swerts, M. (2008). On the role of acting skills for the collection of simulated emotional speech. In *Proceedings of the international conference on spoken language processing (Interspeech 2008)*. Brisbane: Interspeech.

[CR59] Krebs JR, Davies NB (1993). An introduction to behavioural ecology.

[CR60] Laukka P, Audibert N, Aubergé V (2012). Exploring the determinants of the graded structure of vocal emotion expressions. Cognition and Emotion.

[CR61] Laukka, P., Elfenbein, H. A., Chui, W., Thingujam, N. S., Iraki, F. K., Rockstuhl, T., & Althoff, J. (2010). Presenting the VENEC corpus: Development of a cross-cultural corpus of vocal emotion expressions and a novel method of annotating emotion appraisals. In L. Devillers, B. Schuller, R. Cowie, E. Douglas-Cowie, & A. Batliner (Eds.), *Proceedings of the LREC 2010 workshop on corpora for research on emotion and affect* (pp. 53–57). Valletta: European Language Resources Association.

[CR62] Laukka P, Elfenbein HA, Söder N, Nordström H, Althoff J, Chui W, Iraki FK, Thingujam NS (2013). Cross-cultural decoding of positive and negative non-linguistic vocalizations. Frontiers in Psychology.

[CR63] Laukka P, Neiberg D, Forsell M, Karlsson I, Elenius K (2011). Expression of affect in spontaneous speech: Acoustic correlates, perception, and automatic detection of irritation and resignation. Computer Speech and Language.

[CR64] Levenson RW, Ekman P, Davidson RJ (1994). Human emotion: A functional view. The nature of emotion: Fundamental questions.

[CR65] Martin, O., Kotsia, I., Macq, B., & Pitas, I. (2006). The eNTERFACE’05 audio-visual emotion database. In *Proceedings of the 22nd international conference on data engineering workshops, ICDEW’06.* Piscataway, NJ: Institute of Electrical and Electronics Engineers.

[CR66] McKeown G, Valstar M, Cowie R, Pantic M, Schröder M (2012). The SEMAINE database: Annotated multimodal records of emotionally colored conversations between a person and a limited agent. IEEE Transactions on Affective Computing.

[CR67] Morton ES (1977). On the occurrence and significance of motivation-structural rules in some bird and mammal sounds. American Naturalist.

[CR68] Murray IR, Arnott JL (1993). Toward the simulation of emotion in synthetic speech: A review of the literature on human vocal emotion. Journal of the Acoustical Society of America.

[CR69] Neiberg, D., Elenius, K., & Laskowski, K. (2006). Emotion recognition in spontaneous speech using GMMs. In *Proceedings of the 9th international conference on spoken language processing, Interspeech 2006* (pp. 809–812). Pittsburgh, PA: International Speech Communication Association.

[CR70] Nordström, H., & Laukka, P. (2017). *The time course of emotion recognition in speech and music.* Manuscript submitted for publication. 10.1121/1.5108601 31153307

[CR71] Norman, N. (2014). Method acting: ‘Faking it’ is so last year—Commitment is everything. *Newsweek*. Retrived from http://europe.newsweek.com/.

[CR72] Owren MJ, Bachorowski J-A, Coan J, Allen J (2007). Measuring emotion-related vocal acoustics. Handbook of emotion elicitation and assessment.

[CR73] Pell MD, Skorup V (2008). Implicit processing of emotional prosody in a foreign versus native language. Speech Communication.

[CR74] Pittermann J, Pittermann A, Minker W (2010). Handling emotions in human–computer dialogues.

[CR75] Planalp S, Andersen PA, Guerrero LK (1998). Communicating emotion in everyday life: Cues, channels, and processes. Handbook of communication and emotion.

[CR76] Plutchik R (1994). The psychology and biology of emotion.

[CR77] Rosenthal R (1987). Judgment studies: Design, analysis, and meta-analysis.

[CR78] Russell JA (1980). A circumplex model of affect. Journal of Personality and Social Psychology.

[CR79] Russell JA, Bachorowski J-A, Fernandez-Dols JM (2003). Facial and vocal expressions of emotion. Annual Review of Psychology.

[CR80] Scheiner E, Fischer J, Welsch W, Singer W, Wunder A (2011). Emotion expression—The evolutionary heritage in the human voice. Interdisciplinary anthropology: The continuing evolution of man.

[CR81] Scherer KR, Rosenblatt J, Beer C, Busnel M-C, Slater PJB (1985). Vocal affect signalling: A comparative approach. Advances in the study of behavior.

[CR82] Scherer KR (1986). Vocal affect expression: A review and a model for future research. Psychological Bulletin.

[CR83] Scherer KR (2013). Vocal markers of emotion: Comparing induction and acting elicitation. Computer Speech and Language.

[CR84] Scherer KR, Banse R, Wallbott HG, Goldbeck T (1991). Vocal cues in emotion encoding and decoding. Motivation and Emotion.

[CR85] Scherer KR, Bänziger T, Scherer KR, Bänziger T, Roesch EB (2010). On the use of actor portrayals in research on emotional expression. Blueprint for affective computing: A sourcebook.

[CR86] Schuller B, Batliner A, Steidl S, Seppi D (2011). Recognizing realistic emotions and affect in speech: State of the art and lessons learnt from the first challenge. Speech Communication.

[CR87] Sneddon I, McRorie M, McKeown G, Hanratty J (2012). The Belfast induced natural emotion database. IEEE Transactions on Affective Computing.

[CR88] Snowdon CT, Davidson RJ, Scherer KR, Goldsmith HH (2003). Expression of emotion in nonhuman animals. Handbook of affective sciences.

[CR89] Spencer H (1857). The origin and function of music. Fraser’s Magazine.

[CR90] Sundberg J (1998). Expressivity in singing: A review of some recent investigations. Logopedics Phoniatrics Vocology.

[CR91] Sundberg J, Patel S, Björkner E, Scherer K (2011). Interdependencies among voice source parameters in emotional speech. IEEE Transactions on Affective Computing.

[CR92] Tassinary LG, Cacioppo JT (1992). Unobservable facial actions and emotion. Psychological Science.

[CR93] Tatham M, Morton K (2004). Expression in speech: Analysis and synthesis.

[CR94] Truong KP, van Leeuwen DA, de Jong FMG (2012). Speech-based recognition of self-reported and observed emotion in a dimensional space. Speech Communication.

[CR95] van Bezooijen R (1984). Characteristics and recognizability of vocal expressions of emotion.

[CR96] Ververidis D, Kotropoulos C (2006). Emotional speech recognition: Resources, features, and methods. Speech Communication.

[CR97] Visser PS, Krosnick JA, Lavrakas PJ, Reis HT, Judd CM (2000). Survey research. Handbook of research methods in social and personality psychology.

[CR98] Wagner HL (1993). On measuring performance in categorical judgment studies of nonverbal behavior. Journal of Nonverbal Behavior.

[CR99] Wilcox RR (2012). Modern statistics for the social and behavioral sciences: A practical introduction.

[CR100] Williams CE, Stevens KN (1972). Emotions and speech: Some acoustical correlates. Journal of the Acoustical Society of America.

[CR101] Wilting, J., Krahmer, E., & Swerts, M. (2006). Real vs. acted emotional speech. In *Proceedings of Interspeech 2006* (pp. 805–808). Pittsburgh, PA.

[CR102] With S, Kaiser S (2011). Sequential patterning of facial actions in the production and perception of emotional expressions. Swiss Journal of Psychology.

[CR103] Zuckerman M, Larrance DT, Hall JA, DeFrank RS, Rosenthal R (1979). Posed and spontaneous communication of emotion via facial and vocal cues. Journal of Personality.

